# Structure activity relationship of phenolic acid inhibitors of α-synuclein fibril formation and toxicity

**DOI:** 10.3389/fnagi.2014.00197

**Published:** 2014-08-05

**Authors:** Mustafa T. Ardah, Katerina E. Paleologou, Guohua Lv, Salema B. Abul Khair, Abdulla S. Kazim, Saeed T. Minhas, Taleb H. Al-Tel, Abdulmonem A. Al-Hayani, Mohammed E. Haque, David Eliezer, Omar M. A. El-Agnaf

**Affiliations:** ^1^Department of Biochemistry, College of Medicine and Health Science, United Arab Emirates UniversityAl Ain, UAE; ^2^Department of Molecular Biology and Genetics, Democritus University of ThraceAlexandroupolis, Greece; ^3^Department of Biochemistry, Weill Cornell Medical College, Cornell UniversityNew York, NY, USA; ^4^Department of Anatomy, College of Medicine and Health Science, United Arab Emirates UniversityAl Ain, UAE; ^5^College of Pharmacy and Sharjah Institute for Medical Research, University of SharjahSharjah, UAE; ^6^Department of Anatomy, Faculty of Medicine, King Abdulaziz UniversityJeddah, Saudi Arabia; ^7^Faculty of Medicine, King Abdel Aziz UniversityJeddah, Saudi Arabia

**Keywords:** α-synuclein, Parkinson's disease, gallic acid, aggregation, amyloid fibrils, drug discovery

## Abstract

The aggregation of α-synuclein (α-syn) is considered the key pathogenic event in many neurological disorders such as Parkinson's disease (PD), dementia with Lewy bodies and multiple system atrophy, giving rise to a whole category of neurodegenerative diseases known as synucleinopathies. Although the molecular basis of α-syn toxicity has not been precisely elucidated, a great deal of effort has been put into identifying compounds that could inhibit or even reverse the aggregation process. Previous reports indicated that many phenolic compounds are potent inhibitors of α-syn aggregation. The aim of the present study was to assess the anti-aggregating effect of gallic acid (GA) (3,4,5-trihydroxybenzoic acid), a benzoic acid derivative that belongs to a group of phenolic compounds known as phenolic acids. By employing an array of biophysical and biochemical techniques and a cell-viability assay, GA was shown not only to inhibit α-syn fibrillation and toxicity but also to disaggregate preformed α-syn amyloid fibrils. Interestingly, GA was found to bind to soluble, non-toxic oligomers with no β-sheet content, and to stabilize their structure. The binding of GA to the oligomers may represent a potential mechanism of action. Additionally, by using structure activity relationship data obtained from fourteen structurally similar benzoic acid derivatives, it was determined that the inhibition of α-syn fibrillation by GA is related to the number of hydroxyl moieties and their position on the phenyl ring. GA may represent the starting point for designing new molecules that could be used for the treatment of PD and related disorders.

## Introduction

Parkinson's disease (PD) is a neurodegenerative disorder, the incidence of which rises sharply after the fifth decade of life, affecting 1.4 and 3.4% of the population at 55 and 75 years of age, respectively (Wood, 1997). PD affects a region of the brain known as the substantia nigra, resulting in a dramatic loss of dopaminergic neurons and a concomitant plummeting in dopamine levels in striatum. As a consequence, PD is manifested by a plethora of clinical symptoms, with the most prominent being impaired motor activity, muscle rigidity, and resting tremor. Neuropathologically, PD is characterized by the presence of intracellular inclusions known as Lewy bodies (LBs), the main constituent of which are α-synuclein (α-syn) fibrils (Spillantini et al., 1997; El-Agnaf et al., 1998). Moreover, accumulated results over the last few decades show that mitochondrial dysfunction followed by oxidative stress are both associated with neurodegenerative diseases (Leuner et al., 2007; Schmitt et al., 2012).

Although α-syn belongs to the family of natively unfolded proteins, which demonstrate little or no ordered structure, the protein has the intrinsic propensity to fibrillate, giving rise to insoluble fibrils similar to the ones detected in LBs (Hashimoto et al., 1998; Giasson et al., 1999; Conway et al., 2000; Serpell et al., 2000; Greenbaum et al., 2005). Additionally, five missense mutations in the α-syn gene (SNCA), namely A53T (Polymeropoulos et al., 1997), A30P (Krüger et al., 1998), E46K (Zarranz et al., 2004), H50Q (Appel-Cresswell et al., 2013) and G51D (Kiely et al., 2013), have been linked to severe inherited forms of PD, while duplications and triplications of SNCA lead to autosomal dominant PD in a gene dosage-dependent fashion (Singleton et al., 2003; Ibáñez et al., 2004). Moreover, transgenic animal and Drosophila models expressing either wild-type or mutant human α-syn also develop fibrillar inclusions and a Parkinsonian phenotype (Feany and Bender, 2000; Masliah et al., 2000; Kahle et al., 2001; Imai et al., 2011; Cannon et al., 2013). Overexpression of α-syn, especially its mutant forms, is thought to enhance the vulnerability of neurons to DA-induced cell death through an excessive generation of intracellular ROS (Junn and Mouradian, 2002). Many evidences point to the key role of α-syn in the regulation and biosynthesis of DA, the loss of α-syn function as a consequence to its aggregation lead to selective disrupt DA homeostasis and negatively affect dopaminergic neuron survival (Perez et al., 2002). It is noteworthy that the fibrillation of α-syn is implicated in the development of a series of neurodegenerative diseases, including multiple system atrophy (MSA) and dementia with Lewy bodies (DLB), which are collectively referred to as synucleinopathies (Spillantini and Goedert, 2000; Trojanowski and Lee, 2003). Mitochondrial pathophysiology aggressively promotes neuronal dysfunction and loss of synaptic viability, leading ultimately to neurodegeneration (Perier and Vila, 2012), and accumulated evidence from both *in vitro* and *in vivo* studies, postulates a major pathogenic role for a α-syn in mitochondrial dysfunction, thereby providing a link between protein aggregation, mitochondrial damage, and neurodegeneration (reviewed in Camilleri and Vassallo, 2014). Taken together, these findings indicate a central role for α-syn aggregation in PD pathogenesis. α-Syn aggregation proceeds through several key intermediate stages, with monomeric α-syn first assembling into oligomeric forms that gradually generate insoluble amyloid fibrils. Because α-syn aggregation plays a crucial role in PD pathogenesis and related synucleinopathies, intensive effort has been put into identifying compounds that could block or even reverse the aggregation process. Over the years, polyphenols, a set of more than 8000 compounds that contain one or more phenolic rings, have emerged as potent amyloid inhibitors, interfering with the *in vitro* fibril assembly of many amyloidogenic proteins including α-syn, β-amyloid (Aβ), tau-protein and prions (reviewed in Porat et al., 2006).

Gallic acid (GA) is a phenolic acid. Phenolic acids constitute a group of compounds, which are derived from benzoic acid and cinnamic acid, giving rise to hydroxybenzoic acids and hydrocinnamicacids, respectively. GA (3,4,5-trihydroxybenzoic acid) is a benzoic acid derivative that can be found in almost all plants, with the highest GA contents detected in gallnuts, witch hazel, pomegranate, berries such as blackberry and raspberry, sumac, tea leaves and oak bark. GA can also be isolated from the roots of Radix Paeoniae (white-flowered peony), which is commonly used to treat vascular and liver diseases in traditional Chinese medicine (Ho and Hong, 2011). It has been reported that GA possesses anti-oxidant (Kim, 2007), anti-inflammatory (Kroes et al., 1992) and anti-viral (Kreis et al., 1990) properties, and a well-documented anti-cancer activity (Yang et al., 2000; Liu et al., 2011; Ho et al., 2013). Recently, GA has been reported to act as a potent anti-oxidant and free radical scavenger in a rat PD model (Sameri et al., 2011). Additionally, GA was shown to efficiently inhibit α-syn and Aβ aggregation and toxicity *in vitro* (Bastianetto et al., 2006; Di Giovanni et al., 2010).

The aim of the present study was to systematically assess the ability of GA to (a) inhibit α-syn oligomerization and fibrillation, (b) block α-syn-induced toxicity and (c) disaggregate preformed α-syn fibrils. To gain insight of the mechanism of action of GA against α-syn aggregation and toxicity and to establish a structure-activity relationship, we assessed the anti-fibrillogenic effect of eleven different hydroxybenzoic acid derivatives with chemical structures similar to GA. The selection of the phenolic acids was based on the number of the hydroxyl moieties attached to the phenyl ring. To further investigate the role of hydroxyl groups in the inhibitory activity of phenolic acids, we also included and assessed the effect of three different benzoic acid derivatives that have fluorides and methoxy groups instead of hydroxyl moieties.

## Materials and methods

### Expression and purification of recombinant human α-syn

The GST-α-syn fusion construct in pGEX-4T1 vector (kindly provided by Dr. Hyangshuk Rhim, The Catholic University College of Medicine, Seoul, Korea) was inserted into *E.coli* BL21bacteria for protein expression by heat shock transformation. The transformed bacteria were grown in LB medium supplemented with 0.1 mg/ml ampicillin at 37°C in an orbital shaker until the OD600 was 0.5. GST-α-syn expression was then induced by adding 0.5 mM IPTG (Sigma-Aldrich Chemie GmbH, Germany), and the culture was incubated for 2 h at 37°C. The cells were harvested by 15 min centrifugation at 9000 × g (Beckman Coulter, Avanti J-26 XP). The pellets were resuspended in lysis buffer (50 mM Tris-HCl, pH 7.4, 150 mM NaCl, 2 mM EDTA, 1% NP-40, 0.1% DTT), and the lysed pellets were shaken for 10 min at room temperature. The lysed pellets were then subjected to 6 freeze-thaw cycles in liquid nitrogen and a 37°C water bath. The lysates were centrifuged further at 27,000 × g for 15 min. The supernatant was kept for purification with affinity chromatography. Glutathione sepharose 4B beads (Amersham, Sweden) were centrifuged at 500 × g at 4°C for 8 min to remove the storage buffer. The beads were then washed with 20 ml cold PBS followed by spinning at 500 × g at 4°C for 10 min. The cell lysate was mixed with the washed beads and incubated for 1 h at room temperature, followed by centrifugation at 500 × g at 4°C for 8 min. The beads were then washed twice with column washing buffer (50 mM Tris-HCl, 150 mM NaCl, 10 mM EDTA, 1% Triton X-100, pH 8.0), twice with 50 mM Tris-HCl, pH 8.0 and once with 1X PBS. The washed beads were resuspended in 5 ml of 1X PBS. The GST tag, which interacts with glutathione beads, was cleaved by thrombin (1 unit/μl human plasma thrombin, Sigma). The thrombin-catalyzed reaction was incubated overnight at room temperature with continuous mixing. The mixture was then incubated for 5 min at 37°C and centrifuged for 8 min at 500 × g at 4°C. Benzamidinesepharose beads (Amersham, Sweden) were used to remove the thrombin from the solution. Pure α-syn was collected by centrifugation at 500 × g for 8 min at 4°C. The α-syn concentration was estimated using the BCA assay (Pierce Biotechnology, Rockford, IL).

### Aggregation of α-syn *in vitro*

Stock solutions of the tested compounds (10 mM) were prepared in DMSO. Solutions of lower concentrations were prepared by diluting the stock solutions to final concentrations of 25–100μM. The amount of DMSO in the final samples was 1%. Samples of 25μM (unless otherwise stated) α-syn in PBS were aged either alone or with phenolic acids at various molar ratios (phenolic acids to protein molar ratios of 4:1, 2:1, and 1:1). The samples were placed in 1.5 ml sterile polypropylene tubes, sealed with parafilm and incubated at 37°C for 5 days with continuous shaking at 800 rpm in a Thermomixer (Eppendorf). Samples were collected at regular time points. Thioflavin-S (Th-S) fluorescence was measured immediately, while the rest of the samples were stored at −80°C until needed for further analysis.

### Thioflavin-S (Th-S) fluorescence assay

α-Syn fibril formation was monitored by Th-S binding. Th-S is a fluorescent dye that interacts with fibrils in a β-sheet structure. Each sample (10 μl) was mixed with 40 μl of Th-S (25μM) in PBS. Fluorescence was measured in a 384-well, non-treated, black micro-well plate (Nunc, Denmark) using a Victor X3 2030 (Perkin Elmer) microplate reader with excitation and emission wavelengths of 450 and 486 nm, respectively. To allow for background fluorescence, the fluorescence intensity of a blank well containing only PBS solution was subtracted from all readings.

### Transmission electron microscopy (TEM)

Electron microscopy images were produced from α-syn aged in the presence or absence of GA. The samples (5 μL) were deposited on Formvar-coated 400 mesh copper grids, fixed briefly with 0.5% glutaraldehyde (5 μl), negatively stained with 2% uranyl acetate (Sigma-Aldrich) and examined in a Philips CM-10 TEM electron microscope.

### Immunoblotting

Samples (20 ng) of α-syn incubated alone or with GA were mixed with loading sample buffer (250 mM Tris-HCl, pH 6.8, 30% glycerol, 0.02% bromophenol blue) without SDS or boiling and then separated on 1 mm 15% SDS-PAGE gels. The separated proteins were transferred to nitrocellulose membranes (0.45μm, WhatmanGmbh-Germany) at 90 V for 80 min. The membranes were boiled for 5 min in PBS and then blocked for 1 h with 5% non-fat milk prepared in PBS-Tween-20 (0.05% PBST). The membranes were incubated overnight at 4°C with the primary antibody, namely the mouse monoclonal anti-α-syn (211) that recognizes human α-syn (121–125) (Santa Cruz Biotechnology, USA), at a dilution of 1:1000. The membranes were then washed several times with PBST, followed by incubation with HRP-conjugated goat anti-mouse antibody (Dako Ltd., Ely, UK) at a dilution of 1:70,000 for 60 min at room temperature and gentle agitation. The membranes were then extensively washed for 25 min. The immunoreactive bands were visualized with a SuperSignal West FemtoChemiluminescent Substrate Kit (Pierce, Rockford, USA) according to the manufacturer's instructions.

### Immunoassay for measuring oligomeric α-syn

A384-well ELISA plate (NuncMaxisorb,Nunc, Denmark) was coated with 1 μg/ml of non-biotinylated mouse monoclonal anti-α-syn antibody [mAb, 211—recognizes amino acid residues 121–125 of human α-syn (Santa Cruz Biotechnology, California, USA)]diluted in 200 mM NaHCO_3_, pH 9.6 (50 μl/well) and incubated overnight at 4°C. The plate was then washed 4 times with PBST and blocked with 100 μl/well of blocking buffer (5% gelatin from cold water fish skin, 0.05% Tween 20 in 1X PBS pH 7.4) for 2 h at 37°C. After washing 4 times with PBST, 50 μl of the samples were dispensed in each well, and each sample was tested in duplicate. The plates were then incubated at 37°C for another 3 h. After washing 4 times with PBST, 50 μl of biotinylated 211 antibody diluted in blocking buffer to a concentration of 0.4 μg/ml was added and incubated at 37°C for 2 h. The wells were washed 4 times with PBST and incubated with 50 μl/well of Extravidin-Peroxidase (Sigma-Aldrich, GmbH- Germany) diluted 1:7500 in blocking buffer and incubated for 1 h at 37°C. The wells were then washed 4 times with PBST before adding SuperSignal ELISA Femto Maximum Sensitivity Substrate (Pierce Biotechnology, Rockford, USA) (50 μL/well). The chemiluminescence in relative light units was immediately measured using amicroplate reader (Perkin Elmer).

### Culture of BE (2)-M17 human neuroblastoma cells

BE (2)-M17 human neuroblastoma cells were routinely cultured in Dulbecco's MEM/Nutrient Mix F-12 (1:1) (Gibco BRL, Rockville, MD) supplemented with 15% fetal bovine serum and 1% penicillin-streptomycin (100 U/ml penicillin, 100 mg/ml streptomycin). The cells were maintained at 37°C in a humidified incubator with 5% CO2/95% air.

### Cell viability assay

To evaluate the cell viability of BE (2)-M17 human neuroblastoma cells treated with α-syn aggregates, the 3-(4,5-dimethylthiazol-2-yl)-2,5-diphenyltetrazolium bromide (MTT) reduction assay was performed as described previously (Mosmann, 1983). Briefly, BE (2)-M17 cells in DMEM medium were plated at a density of 15,000 cells/100 μl/well in a 96-well plate. After 24 h, the medium was replaced with 200 μ l of OPTI-MEM (Gibco BRL) serum-free medium containing either α-syn aged in the presence or absence of GA or pre-aged α-syn incubated with different concentrations of GA. Samples were diluted in OPTI-MEM to obtain the desired concentration. Cells were then returned into the incubator and incubated for 48 h. A total of 20 μ l of MTT (6 mg/ml) in PBS was added to each well, and the plate was incubated at 37°C for 4.5 h. The medium-MTT solution was removed, cell lysis buffer (100 μ l/well; 15% (w/v) SDS/50% (v/v) N,N-dimethylformamide, pH 4.7) was added, and the plate was incubated overnight at 37°C. Absorbance values at 590 nm were determined using a plate reader (Perkin Elmer).

### Congo red binding assay

Congo red (20μM) was dissolvedin PBS (pH 7.4) and filtered through a 0.45μm filter. Samples of α-syn (5μM), aged alone or with GA at different molar ratios, were mixed with Congo red (final concentration 5μM), and the reaction samples were thoroughly mixed. The UV absorbance spectrum was recorded from 400 to 600 nm in a spectrophotometer (DU-800, Beckman-Coulter) using 10 mm quartz cuvettes (Hellma Analytics-Germany). Monomeric α-syn and Congo red alone were used as negative controls.

### α-syn disaggregation assay

Recombinant α-syn dissolved in sterilized PBS (pH 7.4) was aggregated at a concentration of 25μM as indicated above. Briefly, the α-syn samples were placed in 1.5 ml sterile polypropylene tubes sealed with parafilm and incubated at 37°C for 5 days, with continuous shaking at 800 rpm in a Thermomixer (Eppendorf). The resulting aggregated α-syn was incubated either alone or with GA at various molar ratios (GA: α-syn molar ratios 6:1, 4:1, and 2:1). It should be noted that for the purpose of the experiment, the concentration of α-syn was assumed to be the same as that of the fresh α-syn. The samples were incubated at 37°C for 3 days on a thermomixer with continuous shaking at 800 rpm. Samples were collected at regular time points, and Th-S fluorescence was measured immediately.

### Seeding polymerization assay

The aggregation of monomeric α-syn with or without seeding was carried out as described elsewhere (Di Giovanni et al., 2010). Mature α-syn fibrils were fragmented by sonication to obtain short fibrils, which were employed as ‘seeds’. Briefly, 100μM monomeric α-syn was seeded with 2μMseeds and incubated in the presence or absence of GA (10μM and 50μM) at 37°C for 6 h with continuous shaking. Fibrillation was monitored by Th-S binding as described above.

### Preparing WT-α-syn oligomers

Recombinant α-syn dissolved in sterilized PBS (pH 7.4) was aggregated at a concentration of 100μM. Sample was placed in 1.5 ml sterile polypropylene tubes, sealed with parafilm and incubated at 37°C for 2 days, with continuous shaking at 800 rpm in a Thermomixer (Eppendorf).

### Native page

Samples of α-syn oligomers, α-syn: GA oligomers and α-syn monomers (15 ng), were separated in 3–1% Bis-Tris Native PAGE gel (Novox, Life technologies) according to the manufacturer's instructions. The separated proteins were transferred to PVDF membrane (0.45μm, Thermo Scientific) at 100 V for 120 min, after soaking the methanol for 30 s, and followed by soaking in 1x methanol free transfer buffer for 10 min. The membrane then blocked for 1 h with 5% non-fat milk prepared in PBS-Tween-20 (0.05% PBST). The membrane were incubated overnight at 4°C with the primary antibody, namely the mouse monoclonal anti-α-syn (211) that recognizes human α-syn (121–125) (Santa Cruz Biotechnology, USA), at a dilution of 1:1000. The membranes were then washed several times with PBST, followed by incubation with HRP-conjugated goat anti-mouse antibody (Dako Ltd., Ely, UK) at a dilution of 1:120,000 for 60 min at room temperature and gentle agitation. The membranes were then extensively washed for 25 min. The immunoreactive bands were visualized with a SuperSignal West FemtoChemiluminescent Substrate Kit (Pierce, Rockford, USA) according to the manufacturer's instructions.

### Size exclusion chromatography (SEC) for separating α-syn oligomers and monomers

SEC was carried out using an AKTA FPLC system (GE Healthcare-Sweden) and a superdex 200 column at 4°C, in order to separate the oligomers generated from the aggregation of α-syn with GA (GA: α-syn molar ratio of 4:1). Monomeric α-syn at a concentration of 100μM was aggregated in the presence of GA for 5 days as described above. At the end of the aggregation process, the sample was centrifuged for 45 min at 14,000 × g at 4°C generating a supernatant free from insoluble material. Prior to injecting 80% of the generated supernatant, the column was thoroughly equilibrated with SEC running buffer (1 × PBS, pH 7.4) and the flow rate was set to 0.1 ml/min (0.5 ml/fraction). α-Syn elution was monitored at absorbance wavelengths of 215, 254, and 280 nm. To determine the elution time of monomeric α-syn, molecular weight standards (ferritin 440 kDa, aldolase 171 kDa, abmumin 68 kDa and chymotrypsinogen A 25 kDa) and monomeric α-syn were co-injected into the column and eluted at the same conditions mentioned above. The fractions eluting between 7-9 ml CV were combined and labeled as oligomers (sample P1), whereas the fractions eluting in the 13–15 ml CV were combined and labeled as monomers (sample P2). The P1 and P2 fractions were further characterized by western blotting and TEM.

### UV scanning

The P1 and P2 samples, representing the oligomeric and monomeric fractions of SEC, respectively, were concentrated using a speed vac (CentriVap, Labconco). Their protein content concentration was estimated by the BCA assay. The UV absorbance spectrum was recorded from 200 to 600 nm in a spectrophotometer (DU-800, Beckman-Coulter) using 10 mm quartz cuvettes (Hellma Analytics-Germany) and employing equal concentrations of both P1 and P2. Fresh monomeric α-syn was used as negative control.

### NMR

For NMR studies, recombinant 15N-labeled α-syn was expressed and purified as previously described (Eliezer et al., 2001; Bussell and Eliezer, 2003), resuspended in PBS at pH 6.5. Two-dimensional proton-nitrogen correlation spectra were acquired for α-syn at 200μM concentration in the absence of GA and in the presence of increasing GA: α-syn stoichiometries of 0.5:1, 1:1, 2:1, 5:1, and 10:1. Data were collected on a Varian 600 MHz Unity Inova spectrometer equipped with a cold probe.

## Results

### The effect of GA on α-syn fibril formation

**Figure 1:**
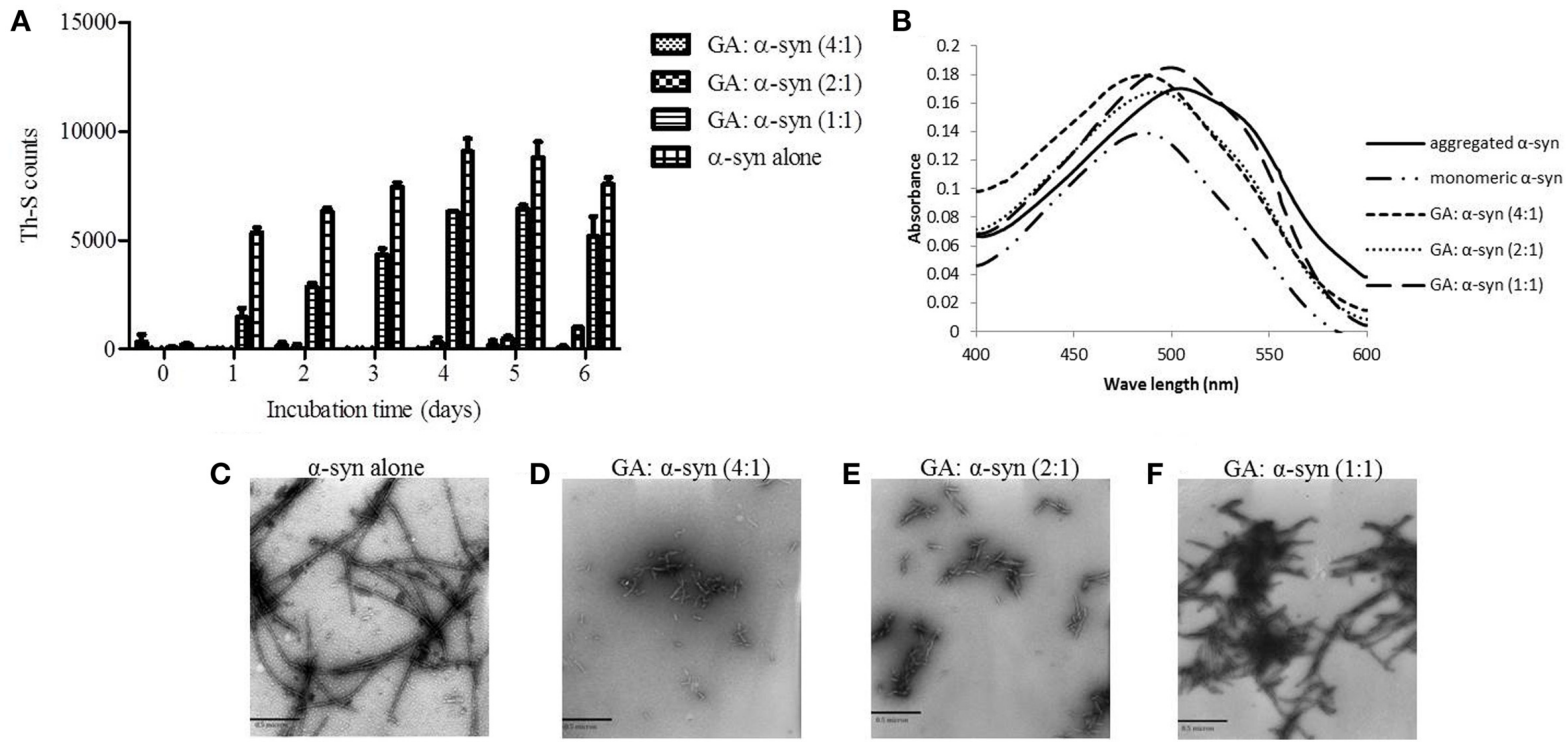
**GA inhibits** α-**syn fibrillation in a concentration-dependent manner. (A)** Samples of α-syn (25μM) were incubated alone or in the presence of GA (molar ration of GA: α-syn 4:1, 2:1, 1:1) for 6 days with continuous shaking at 37°C. Fibril formation was estimated by Th-S fluorescence. The assay was performed in triplicate (average of triplicate measurement ± standard deviation). **(B)** Congo red binding for samples of α-syn (25μM) incubated alone or in the presence of GA (molar ratio of GA: α-syn 4:1, 2:1, 1:1) for 6 days with continuous shaking at 37°C. Samples of α-syn (5μM) aged alone or with GA were mixed with Congo red at a final concentration of 5μM. The reaction samples were thoroughly mixed and placed in a 10 mm quartz cuvette. The UV absorbance spectra were recorded from 400 to 600 nm. C-F. Electron microscopy images of negatively stained samples of α-syn (25μM) aged alone or in the presence of GA (molar ratio of GA: α-syn 4:1, 2:1, 1:1) for 6 days with continuous shaking at 37°C. **(C)** Aged α-syn alone. **(D)** α-Syn aged in the presence of GA at a molar ratio of GA: α-syn 4:1. **(E)** α-Syn aged in the presence of GA at a molar ratio of GA: α-syn 2:1. **(F)** α-Syn aged in the presence of GA at a molar ratio of GA: α-syn 1:1. Scale bar, 500 nm.

To investigate the effect of GA on α-syn fibrillation, 25μM of α-syn was incubated with GA at varying molar ratios of GA: α-syn of 4:1, 2:1, and 1:1 for a period of 6 days. GA inhibited the formation of α-syn fibrils in a concentration-dependent manner as indicated by the Th-S assay (Figure 1A). Taking into account the lack of Th-S signal at the 4:1 ratio, GA exhibited an excellent inhibitory effect on α-syn fibrillation, inhibiting it completely during the 6-day incubation (Figure 1A). At a 2:1 ratio, GA also suppressed the formation of fibrils to a great extent, and the Th-S signal was detected only after 4 days of incubation, while after 6 days, GA reduced the Th-S counts to approximately one fifth of the control counts (Figure 1A). Even at a 1:1 ratio, GA hindered the fibrillation of α-syn but to a smaller extent than the other ratios (Figure 1A). The anti-fibrillogenic activity of GA was further assessed by the Congo red binding assay. Congo red (CR) is a dye with high affinity for amyloid fibrils. The absorption maximum of CR alone (5μM) was 490 nm, while when incubated with α-syn amyloid fibrils, the absorption maximum shifted to 508 nm (Figure 1B). This shift in the peak absorption wavelength represents the binding of CR to the β-sheet-rich fibrils. However, this prominent shift was not observed for the fresh α-syn sample or when α-syn was aged in the presence of GA (Figure 1B), indicating that GA inhibited the formation of structures with a β-sheet conformation. In fact, GA blocked the formation of β-sheets in a dose-dependent manner, similar to the Th-S results. It was observed that the lower the GA concentration, the more prominent the peak absorbance wavelengths shift was (485, 493, and 500 nm for 100, 50, and 25μM GA, respectively). Electron microscopy images also confirmed that GA not only affected the extent of fibrillation in a dose-dependent fashion but also the morphology of α-syn fibrils. Indeed, instead of forming dense meshes of long fibrils (longer than 1μm) as observed in aged α-syn alone (Figure 1C), α-syn aged in the presence of 100μM (Figure 1D) and 50μM of GA (Figure 1E) generated thin, sheared fibrils that were approximately 0.1–0.2μm in length. α-Syn aged in the presence of 25μM GA (Figure 1F) formed longer fibrils (approximately 0.5μm long), which were arranged in dense networks.

### The effect of GA on α-syn oligomerization (early aggregates)

**Figure 2:**
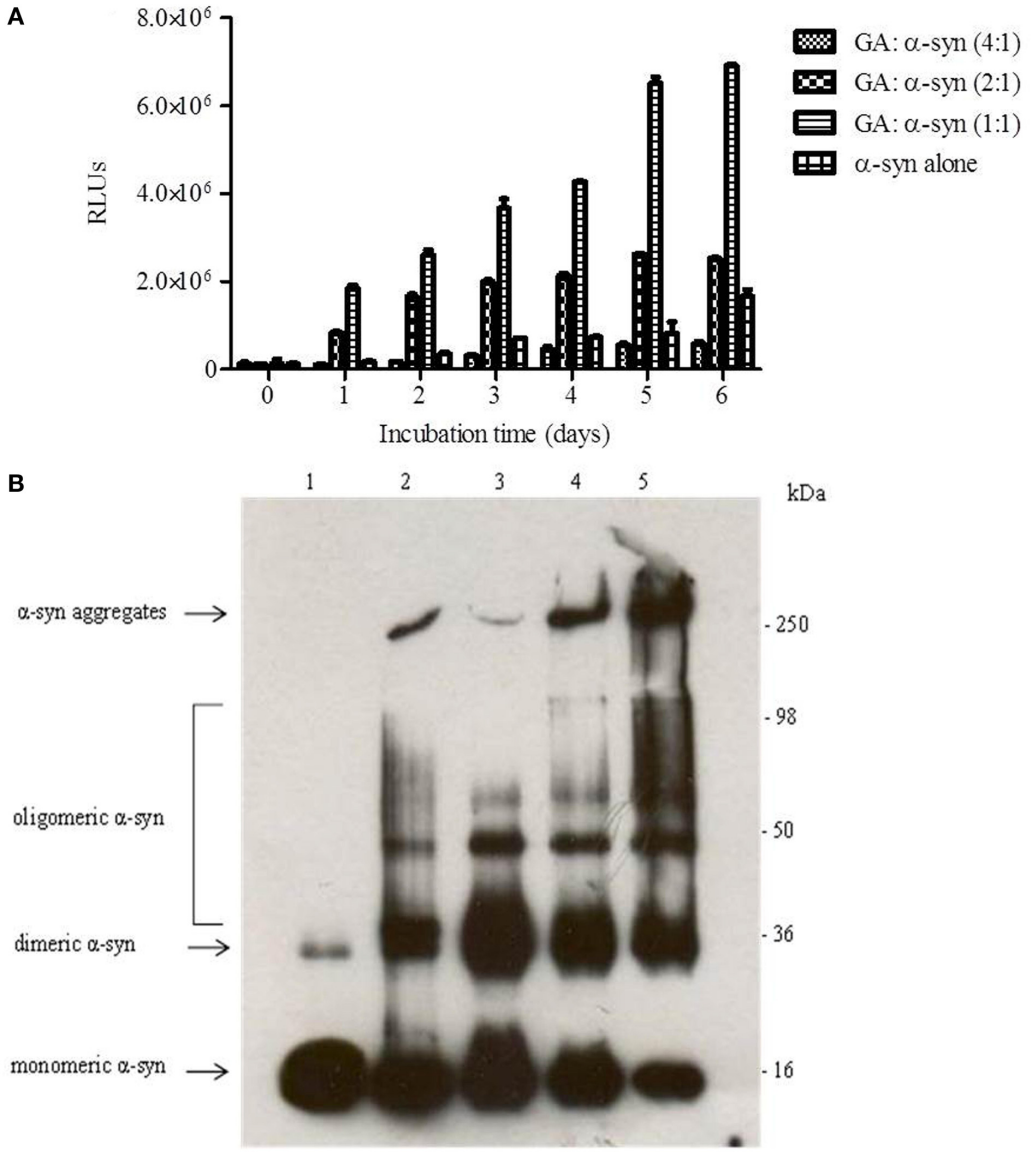
**GA inhibits α-syn oligomerization at a high concentration, but at lower concentrations it promotes oligomerization. (A)** Samples of α-syn (25μM) aged alone or in the presence of GA at different molar ratios for 6 days with continuous shaking at 37°C were assessed for their ability to inhibit the formation of oligomers by the oligomeric ELISA assay. The assay was performed in duplicate (average of duplicate measurements ± standard deviations). **(B)** Immunoblot analysis of the effect of GA on α-syn oligomerization. Fresh or aged α-syn samples alone or in the presence of GA at molar ratios of GA: α-syn 1:1, 2:1 and 4:1 incubated for 6 days with continuous shaking at 37°C were separated by electrophoresis in a15% SDS-PAGE gel. Lane 1: fresh α-syn; lane 2: aged α-syn; lane 3: GA: α-syn molar ratio of 4:1, lane 4: GA:α-syn molar ratio 2:1 and lane 5: GA: α-syn molar ratio 1:1.

To evaluate the effect of GA on α-syn oligomer formation, samples of α-syn aged alone or in the presence of GA at different molar ratios were assessed for their oligomeric content by oligomer-specific ELISA and immunoblotting. For the detection of α-syn oligomeric species in the samples, we employed a novel ELISA developed in our laboratory (El-Agnaf et al., 2006), which specifically recognizes the oligomeric species present in the samples. The ELISA results (Figure 2A) indicated that GA could only inhibit the formation of α-syn oligomers when employed at the highest concentration, namely 100μM. Interestingly, at lower concentrations, GA appeared to enhance α-syn oligomerization compared to the control (Figure 2A). The samples of α-syn aged in the presence of 25 and 50μM GA exhibited gradually increasing oligomeric content, which was much higher than the control (Figure 2A).

Similarly, immunoblotting of the same samples (Figure 2B) indicated a decrease in the oligomeric species when α-syn was aged with 100μM GA (Figure 2B), while there was a characteristic increase in the band corresponding to dimeric α-syn (Figure 2B). The same sample was also characterized by the presence of strong monomeric and trimeric (approximately 50 kDa) bands and the absence of the bands corresponding to larger α-syn aggregates with high molecular weight. However, in the presence of 50 and 25μM GA, the bands corresponding to oligomeric species were much stronger, with a concentration-dependent decrease in the bands corresponding to monomeric, dimeric and trimeric species. Additionally, the intensity of the band corresponding to larger α-syn aggregates increased in a dose-dependent manner. These data indicate that GA at low concentrations may have the ability to stabilize α-syn oligomers.

### The effect of GA on preformed α-syn amyloid fibrils

**Figure 3:**
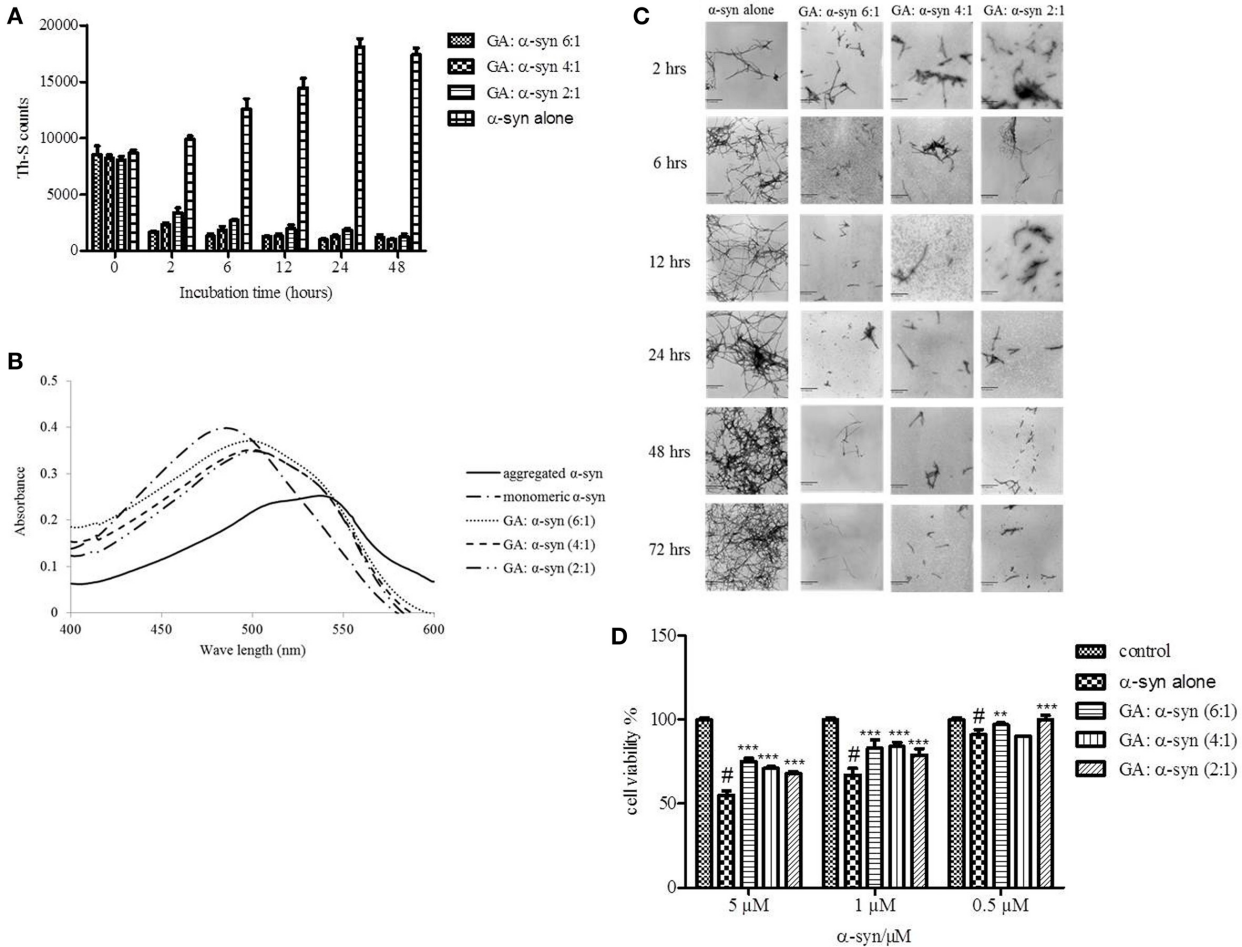
**GA disaggregates preformed α-syn fibrils in a concentration-dependent manner. (A)** Samples of aggregated α-syn were incubated for 48 h at 37°C in the absence or presence of various concentrations of GA (GA: α-syn 6:1, 4:1, 2:1). The fibril content was then measured by the Th-S binding assay. The assays were performed in triplicate (average of triplicate measurements ± standard deviations). **(B)** Congo red binding to samples of pre-aged α-syn (25μM) incubated alone or in the presence of GA (molar ratio of GA: α-syn 6:1, 4:1, 1:1) for 72 h with continuous shaking at 37°C. Samples of α-syn (5μM) incubated alone or with GA at different molar ratios were mixed with Congo red, at a final concentration of 5μM. The reaction samples were thoroughly mixed and placed in a 10 mm quartz cuvette. The UV absorbance spectra were recorded from 400 to 600 nm. **(C)** Electron microscopy images of negatively stained samples of pre-aged α-syn (25μM) incubated alone or in the presence of GA for 72 h with continuous shaking at 37°C. 1. α-Syn aged alone. 2. α-Syn aged in the presence of GA at a GA: α-syn molar ratio of 6:1. 3. α-Syn aged in the presence of GA at a GA: α-syn molar ratio of 4:1. 4. α-Syn aged in the presence of GA at a GA: α-syn molar ratio of 2:1. *Scale bar*, 500 nm. **(D)** The disaggregation of preformed α-syn fibrils by GA generated species that were less toxic to the cells. The viability of BE (2)-M17 human neuroblastoma cells was assessed by the MTT assay. The results are expressed as percentages of the control average (i.e., untreated cells). The α-syn species generated by 72 h incubation of preformed α-syn fibrils in the presence or absence of GA were added to the cells 48 h prior to MTT addition (average of 3 wells ± SD. Statistical analysis was performed using two tailed unpaired *t*-test, ^***^p < 0.001; ^**^p < 0.01).

Given that GA was shown to be such an effective inhibitor of α-syn fibrillation, we investigated whether it could also reverse fibrillation. Therefore, 25μM of preformed α-syn fibrils were incubated at 37°C in the presence of GA at molar ratios of GA: α-syn of 6:1, 4:1, and 2:1 for a period of 48 h. By measuring the Th-S fluorescence counts (Figure 3A), we estimated the fibril content at the indicated time points. At time 0, the Th-S counts were approximately 8000 for all samples (Figure 3A). The α-syn fibrils that were incubated alone continued to aggregate further, as indicated by the increase in Th-S counts (Figure 3A). However, the α-syn fibrils that were incubated in the presence of GA disaggregated over time as shown by the decrease in the Th-S counts (Figure 3A). It is noteworthy that after 24 h of incubation, α-syn fibrils incubated without GA gave approximately 18,000 Th-S counts, while the fibrils incubated with all tested concentrations of GA produced less than 2000 Th-S counts (Figure 3A). Thus, GA disaggregated preformed α-syn fibrils in a dose-dependent fashion.

After 48 h of incubation, the samples were also tested for their CR binding (Figure 3B). As expected, α-syn fibrils incubated in the presence of GA produced spectra with a peak absorbance wavelength that was slightly shifted compared to the controls, indicating minimal β-sheet content. In contrast, the fibrils of α-syn that were incubated alone produced a peak absorbance at 550 nm, indicating a high β-sheet content (Figure 3B). These findings were confirmed by EM (Figure 3C). All samples at time 0 were characterized by the presence of dense meshes of fibrils longer than 0.5μm. However, the α-syn fibrils incubated in the presence of GA gradually became thinner and shorter and appeared fragmented, isolated and scarce, unlike the control, which continued to demonstrate networks of long fibrils (Figure 3C). Furthermore, similar results were obtained when the experiment was performed using α-syn fibrils after reaching plateau in Th-S assay (Figure S1).

To assess whether GA disaggregation of α-syn fibrils was accompanied by a decrease in α-syn toxicity, we then evaluated the toxicity of the α-syn species that resulted from the disaggregation experiment on human neuroblastoma M17 cells (Figure 3D). The samples were diluted to final α-syn concentrations of 0.5, 1, and 5μM. Preformed α-syn fibrils incubated for 6 days in the absence of GA decreased cell viability in a dose-dependent manner. However, preformed α-syn fibrils incubated with GA for 6 days generated species that were less toxic compared to the control. This trend was most prominent for 5μM α-syn, which, when incubated in the absence of GA, induced death of 45% of the cells, but in the presence of GA, cell death was less than 25%.

### The effect of GA on the seeding of α-syn aggregation

**Figure 4:**
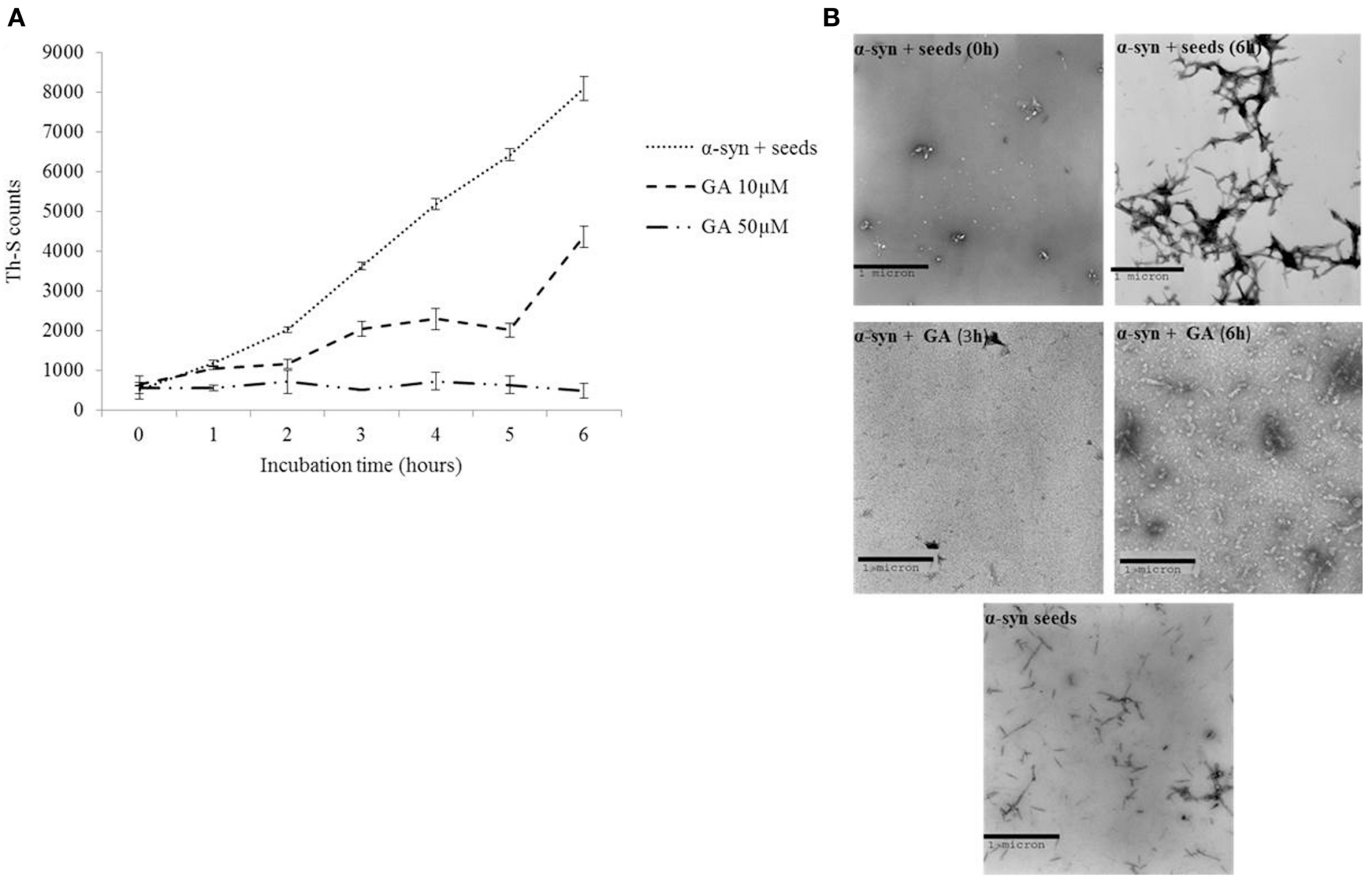
**GA inhibits the seeded fibrillation of α-syn. (A)** Samples of α-syn (100μM) seeded with short fibrillar α-syn (2μM) were incubated in the presence or absence of GA at different concentrations (10–50μM) for 6 h with continuous shaking at 37°C. The extent of fibrillation was estimated by the Th-S binding assay. The assays were performed in triplicate (average of triplicate measurements ± standard deviations). **(B)** Electron microscopy images of negatively stained samples of seeds alone and α-syn+ seeds incubated alone or in the presence of GA (50μM) for 6 h with continuous shaking at 37°C. *Scale bar*, 1000 nm.

It has been previously shown that the process of amyloid fibril formation follows a nucleation-dependent polymerization (Jarrett and Lansbury, 1992). According to this model, soluble species generated via the nucleation of oligomeric species (nucleation or lag time phase), which in turn polymerize (polymerization or growth phase) to generate fibrils, reaching thus a final plateau known as the equilibrium phase (Harper et al., 1999). Small aggregates or seeds have been shown to accelerate the nucleation phase of amyloid formation both *in vitro* and *in vivo* by a process known as seeding (Jarrett and Lansbury, 1993; Harper and Lansbury, 1997; Volpicelli-Daley et al., 2011; Luk et al., 2012). Given that GA inhibited both α-syn fibrillation and disaggregated preformed α-syn fibrils, we sought to identify the effect of this phenolic acid on the seeding of α-syn aggregation. More specifically, mature α-syn fibrils were fragmented by sonication to obtain short fibrils, which were employed as “seeds” (Figure 4B). These short fibrillar “seeds” were then added to monomeric α-syn, which was allowed to aggregate as described above. As expected, the addition of short fibrillar seeds accelerated the fibrillation of α-syn as indicated by the increased Th-S counts. Indeed, with seeding, the extent of α-syn fibrillation after 6 h of incubation was comparable to the fibrillation of the protein incubated for 72 h without seeding (Figures 1, 4B, respectively).

In order to assess the effect of GA on the seeding of α-syn aggregation, 10 and 50μM GA was added to 100μM monomeric α-syn containing seeds at a final concentration of 2μM, and the mixture was incubated with continuous mixing at 37°C for 6 h. GA at 50μM inhibited the seeded fibrillation of α-syn by approximately 90% as indicated by the extremely low Th-S counts. At lower concentrations (10μM), GA also had an inhibitory effect on the seeded fibrillation of α-syn but to a smaller extent. At 10μM, GA inhibited seeded fibrillation by 40–50% (Figure 4A). These findings were confirmed by TEM (Figure 4B).

### The effect of GA on α-syn aggregates-induced toxicity

**Figure 5:**
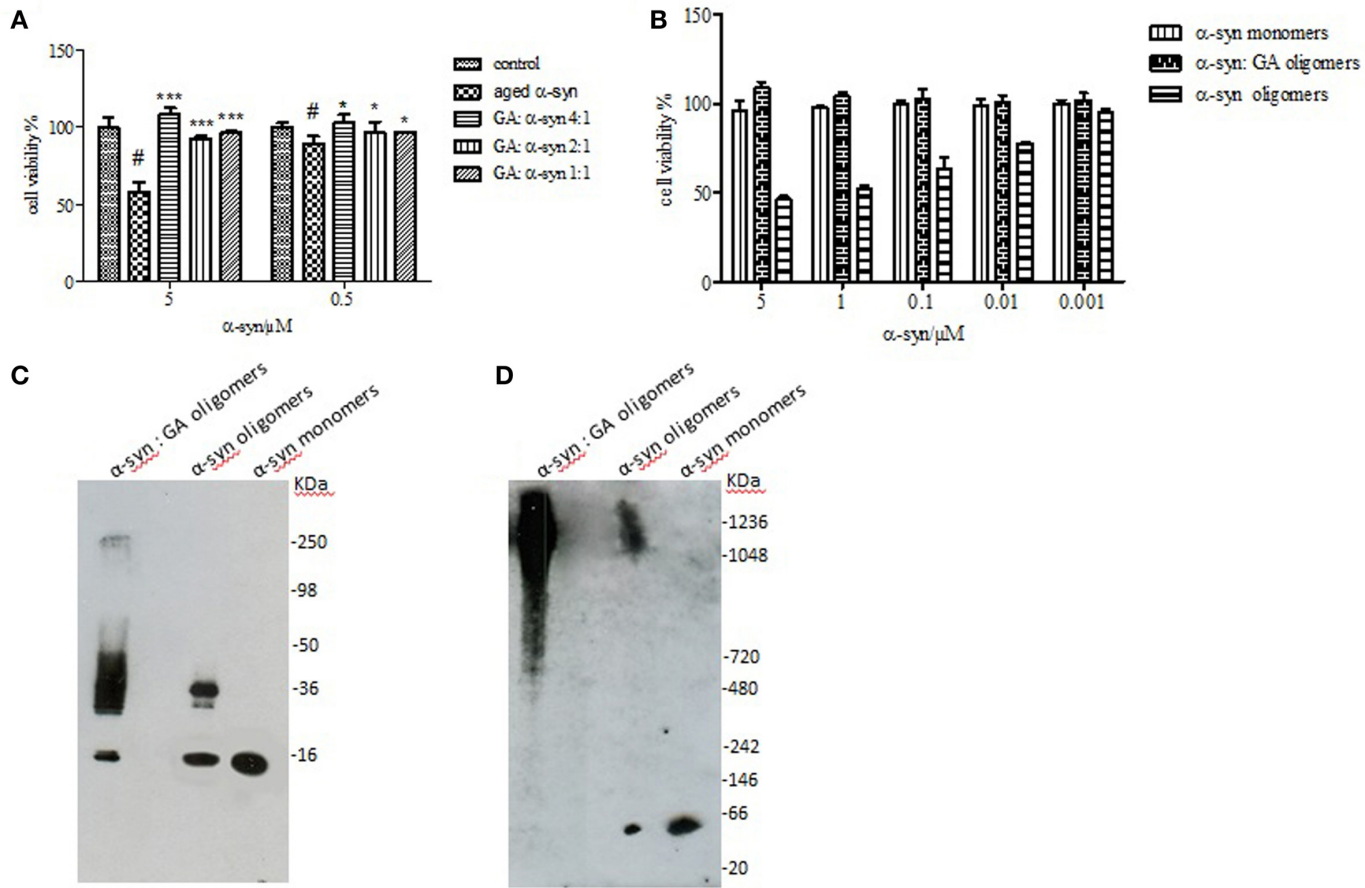
**Effect of GA on the toxicity of aggregated α-syn. (A)** The viability of BE (2)-M17 human neuroblastoma cells was estimated by the MTT assay. The results are expressed as percentage of the control average (i.e., untreated cells). The cells were treated with aggregated α-syn with/without GA for 48 h prior to MTT addition (average of 3 wells ± standard deviation. Statistical analysis was performed using two-tailed unpaired *t*-test, ^***^, *p* < 0.001; ^*^, *p* < 0.05). **(B)** The viability of BE (2)-M17 human neuroblastomacells was estimated by the MTT assay. The results are expressed as percentage of the control average (i.e., untreated cells). The cells were treated with α-syn oligomers in absence or presence of GA for 48 h prior to MTT addition (average of 3 wells ± standard deviation. **(C)** Immunoblot analysis of α-syn oligomers generated in presence or absence of GA, separated by electrophoresis in a 15% SDS-PAGE gel. **(D)** Immunoblot analysis of α-syn oligomers generated in presence or absence of GA, separated by electrophoresis in a 3–12% Native-PAGE gel.

It has been reported that the oligomeric intermediates are the neurotoxic species in the amyloid fibrillation pathway (Allsop et al., 2001; El-Agnaf et al., 2001, 2006; Vendruscolo et al., 2001; Walsh et al., 2002; Argyriou et al., 2012; Breydo et al., 2012; Colla et al., 2012). As described above, at high concentration (4:1), GA inhibited both early and late aggregate formation, whereas at low concentrations (2:1 and 1:1), it stabilized α-syn oligomers (Figure 2A). To determine the effect of GA on toxicity conferred by α-syn aggregates, a cell-based toxicity assay, the MTT assay, was conducted with human neuroblastoma M17 cells. The samples were diluted to final α-syn concentrations of 5 and 0.5μM. The MTT assay showed that α-syn aged in the absence of GA decreased cell viability in a dose-dependent manner (Figure 5). However, when α-syn was aged in the presence of a high concentration of GA (4:1), there was visible neuroprotection of the cells observed (Figure 5). At low concentrations (2:1 and 1:1), GA exhibited a minor protective effect against α-syn toxicity, possibly due to the increase in oligomers as demonstrated by the ELISA results and the immunoblotting analysis (Figures 2A,B, respectively). Our results clearly demonstrate that GA reduced the toxicity of α-syn at a high concentration (100μM) by significantly inhibiting the formation of toxic α-syn oligomers. The toxicity of the oligomers resulted from α-syn: GA 1:4 was compared with α-syn oligomers prepared in the absence of GA as described in materials and methods section by MTT assay (Figure 5B). Both samples were characterized for the presence of oligomers by SDS PAGE (Figure 5C) and native PAGE (Figure 5D). It was clear that the oligomers resulting from the incubation of α-syn with GA were not toxic comparing with oligomers in absence of GA. Moreover, these oligomers were stable under denaturing conditions (Figure 5C) compared those formed in the absence of GA.

### GA inhibition of α-syn fibrillation is mediated via binding to the intermediate species and forming stable oligomers

**Figure 6:**
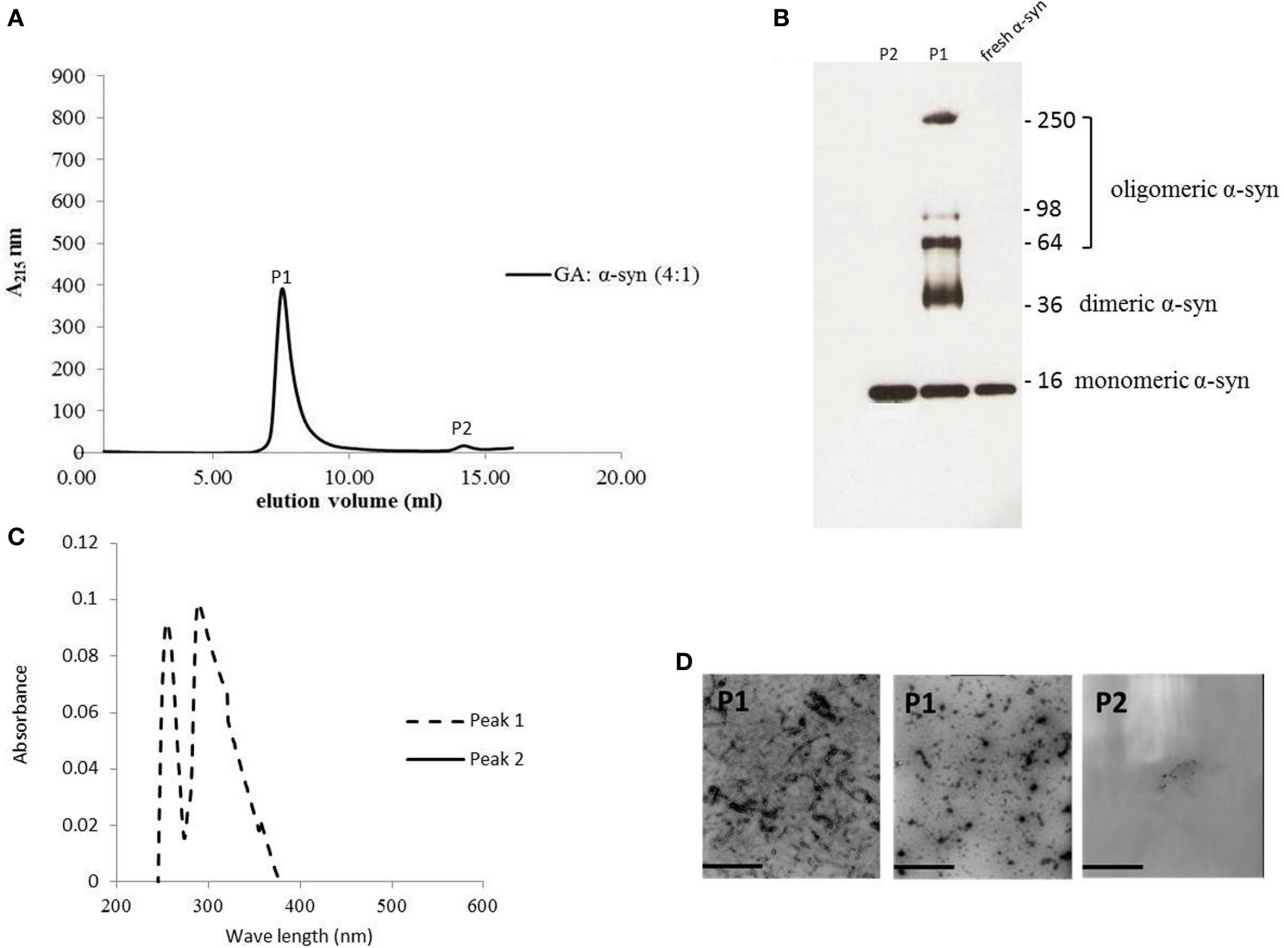
**GA binds to α-syn oligomers (GA: α-syn molar ratio of 4:1). (A)** Gel filtration profile of the 5-day-aggregated α-syn in the presence of GA at a GA: α-syn molar ratio 4:1 (α-syn concentration = 100μM) using a superdex 200 SE column. P1 sample contains the isolated fractions corresponding to the oligomeric peak and P2 the isolated fractions corresponding to the monomeric peak. The elution was monitored at the absorbance wavelength of 215 nm. **(B)** Immunoblot analysis of the samples P1 and P2 separated by electrophoresis in a 15% SDS-PAGE gel. **(C)** UV absorbance spectra of samples P1 and P2. The UV absorbance was recorded between 200 and 600 nm employing a 10 mm quartz cuvette. **(D)** Electron microscopy images of negatively stained samples P1 and P2 of α-syn in the presence of GA (molar ratio of GA: α-syn 4:1) purified by SEC. *Scale bar*, 1000 nm.

**Figure 7:**
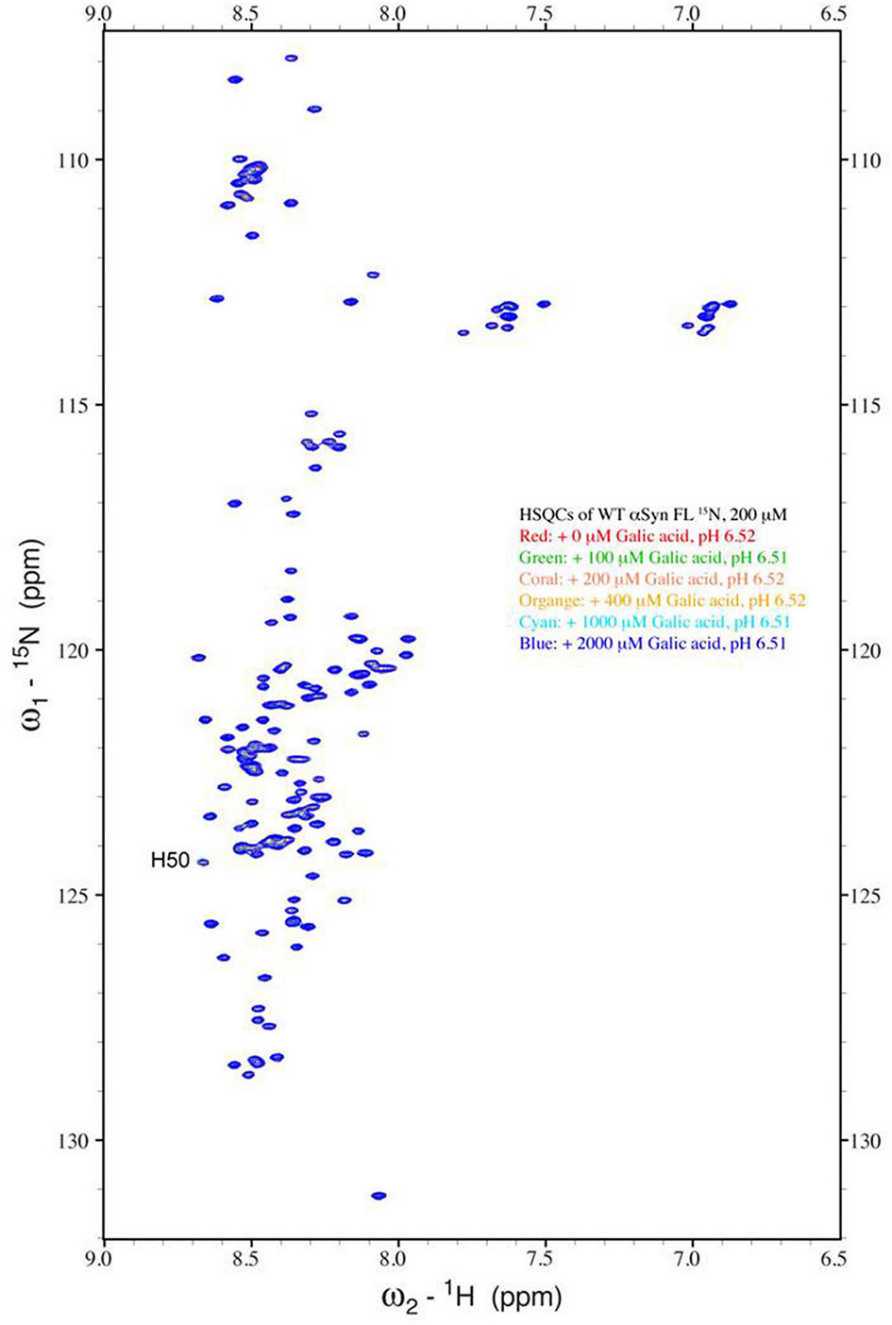
**Analysis of GA binding to monomeric α-syn by NMR spectroscopy**. Proton-Nitrogen correlation (HSQC) spectra of monomeric α-syn in the presence of increasing ratios of GA: α-syn demonstrating that there are no significant changes in the positions of the NMR resonances, indicating the lack of an interaction between GA and monomeric α-syn. Protein concentration was ca. 200μM.

The strong inhibitory effect that GA exerted on fibrillation together with the stimulating effect it had on α-syn oligomerization at lower GA concentrations, prompted us to investigate further the interaction of GA with α-syn oligomers. For this purpose, monomeric α-syn (100μM) was aggregated in the presence of GA (GA: α-syn 4:1). After 5 days of incubation the samples were centrifuged and the supernatant was injected in a superdex 200 SE column. The elution volume for monomeric α-syn was determined by molecular weight standards (Figure S2A), and was eluted in a peak corresponding to column volume of 13–15 mL (Figure S2B), while oligomeric α-syn eluted in a peak corresponding to column volume of approximately 7–9 ml (Figure 6A). The fractions corresponding to the oligomeric and monomeric α-syn peaks were separately pooled together giving rise to P1 and P2 samples, respectively (Figure 6A), which were concentrated using a speed vac. The α-syn species in samples P1 and P2 were characterized by western blotting (Figure 6B). According to the western blotting results, the oligomers generated during the incubation of α-syn with GA are stable under denaturing conditions (Figures 5C, 6B). Electron microscopy of the same samples indicates the presence of different species of oligomers in P1 (Figure 6D), in agreement with the imunoblotting results (Figure 6B). In order to detect the incorporated GA in the P1 and P2 samples, we exploited the property of GA to produce UV absorbance spectra with two peaks, one at 225 nm and one at 260 nm. In the sample containing GA: α-syn at 4:1 molar ratio, we detected GA only in the oligomeric P1 samples (Figure 6C). These findings strongly support the hypothesis that GA binds to the oligomeric species and stabilizes them. To further evaluate whether GA interacts with α-syn monomers, we monitered a titration of GA into a solution of monomeric α-syn using two-dimensional NMR spectroscopy, which provides signals covering the entire amino acid sequence of α-syn (Figure 7). At stoichiometries of up to 10:1 GA: α-syn we observed no significant chemical shift or resonance intensity changes (Figures S3A–E), confirming that GA does not interact significantly with monomeric α-syn. This result is largely in agreement with NMR studies of related compounds Entacapone, Tolcapone and Quercetin (Di Giovanni et al., 2010).

### Investigating the structure-activity relationship of GA inhibition of α-syn fibrillation

**Figure 8:**
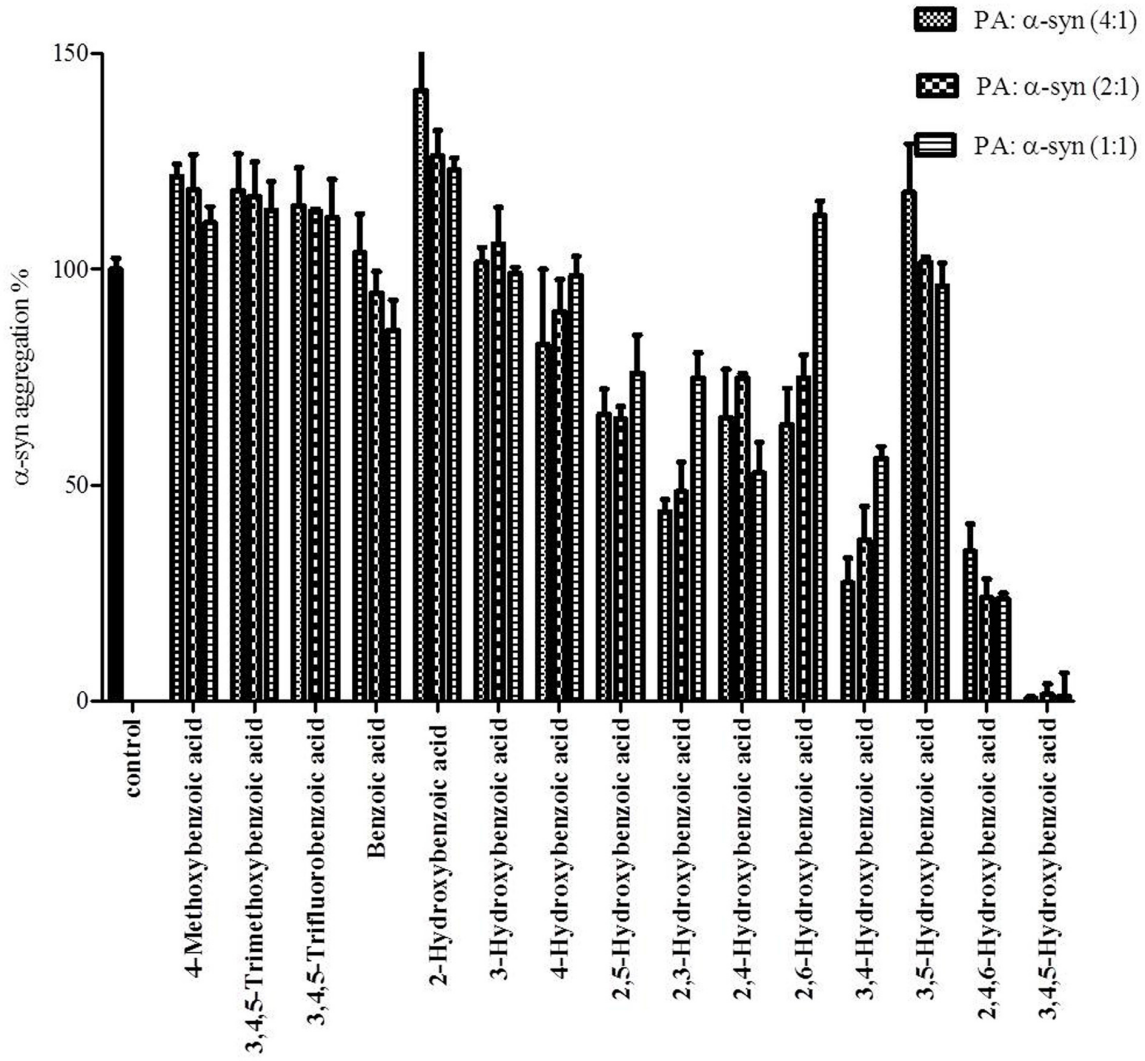
**Effect of different benzoic acid derivatives (phenolic acids, PA) and the effect of methoxy and fluoro groups in benzoic acid derivatives on α-syn fibrillation**. Samples of α-syn (25μM) were incubated alone or in the presence of different benzoic acid derivatives (PA: α-syn molar ratios of 4:1, 2:1, 1:1) for 6 days with continuous shaking at 37°C. The fibril formation was measured by the Th-S binding assay and expressed as a percentage of the fibril content of α-syn aged alone. The assay was performed in triplicate (average of triplicate measurements ± standard deviations).

To define the most effective molecular scaffolds against α-syn fibril formation and establish a structure-activity relationship for GA, we exploited the structural diversity of benzoic acid derivatives (i.e., hydroxybenzoic acids). Twelve hydroxybenzoic acids, including GA, salicylic acid and gentisic acid (see Table 1) were assessed for their anti-fibrillogenic properties by the Th-S binding assay (Figure 8). The structure of the tested hydroxybenzoic acids is characterized by the presence of a phenyl ring that has a carboxyl group and hydroxyl moieties (OH) attached to the ring at different positions. The selection of the compounds examined in the present study was based on the number (between 0 and 3) and position of the hydroxyl groups attached to the phenyl ring (see Table 2).

**Table 1:** **Description and names of the tested benzoic acid derivatives**.

**Name of compound**	**MW**
Benzoic acid	122.12
2-Hydroxybenzoic acid (salicylic acid)	138.12
3-Hydroxybenzoic acid	138.12
4-Hydroxybenzoic acid	138.12
2,3-dihydroxybenzoic acid	154.12
2,4-dihydroxybenzoic acid	154.12
2,5-dihydroxybenzoic acid (gentisic acid)	154.12
2,6-dihydroxybenzoic acid	154.12
3,4-dihydroxybenzoic acid	154.12
3,5-dihydroxybenzoic acid	154.12
2,4,6-trihydroxybenzoic acid	188.13
3,4,5-trihydroxybenzoic acid (GA)	170.12
3,4,5-trihfluorobenzoic acid	176.09
3,4,5-trimethoxybenzoic acid	212.20
4-methoxybenzoic acid	152.15

**Table 2:**
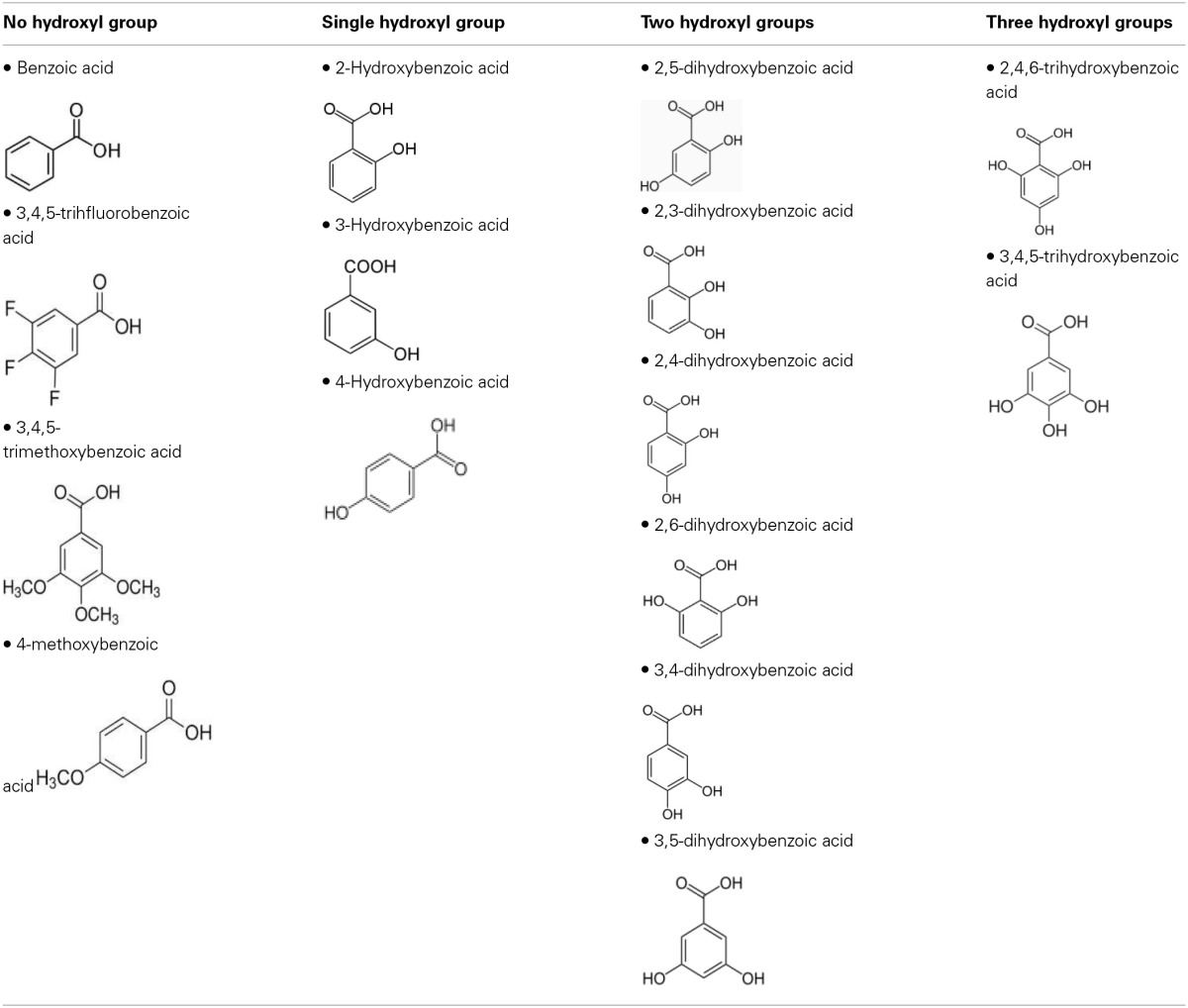
**The compounds were divided into four groups based on the number of hydroxyl groups attached to the phenyl ring**.

The kinetics of α-syn fibrillation was studied over a period of 5 days in the presence or absence of the phenolic acids. Based on the Th-S assay results (Figure 8 and Table 3), the effect of the twelve phenolic acids on α-syn fibrillation varied greatly. The extent of the inhibition of α-syn fibrillation was ~ 99% for GA, ~ 72% for 2,4,6-trihydroxybenzoic acid, ~ 60% for 3,4-dihydroxybenzoic acid, ~ 30% for five compounds including 2,6-dihydroxybenzoic acid and 4-hydroxybenzoic acid and only 5% for benzoic acid (see Figure 8 and Table 3). Moreover, three compounds, 2-hydroxybenzoic acid (salicylic acid), 3-hydroxybenzoic acid and 3,5-dihydroxybenzoic acid, failed to inhibit α-syn fibrillation, with salicylic acid appearing to enhance the aggregation of α-syn compared to the control (see Figure 8 and Table 3). By comparing the structures of the most effective compounds and the least effective ones, we conclude that better inhibition of fibrillation is observed when there are a large number of hydroxyl groups (-OH) attached to the phenyl ring of the compound. Moreover, the positions at which the hydroxyl groups are attached to the phenyl ring also appear to play a critical role. GA has hydroxyl groups attached at positions 3 and 5 (see Table 2) and is much more effective than 2,4,6-trihydroxybenzoic acid, which also bears three -OH groups but at different positions (Figure 8). Interestingly, 3,4-dihydroxybenzoic acid, which only lacks an -OH group at position 5 compared to the very effective GA, is the third best inhibitor of α-syn fibrillation (Figure 8). Among all the dihydroxybenzoic acids tested, the ones with -OH groups at two consecutive positions, i.e., 3,4-dihydroxybenzoic acid (60% inhibition) and 2,3-dihydroxybenzoic acid (44% inhibition), are more potent inhibitors compared to those that have -OH groups at non-consecutive positions, i.e., 2,5-dihydroxybenzoic acid (30% inhibition) and 3,5-dihydroxybenzoic acid (0% inhibition) (Figure 8). Additionally, all compounds with one -OH group, as well as the benzoic acid without a -OH group, failed to show any inhibitory effect on α-syn aggregation (Figure 8).

**Table 3:** **Summary of Th-S results showing the percentage of inhibition of fibril formation comparing with number of OH groups around the phenyl ring**.

**Symbol**	**Compound**	**OH group**	**Inhibition % of α-syn aggregation**
PA1	3,4,5-trihydroxybenzoic acid	3	99
PA2	2,4,6-trihydroxybenzoic acid	3	72
PA3	2,3-dihydroxybenzoic acid	2	44
PA4	2,4-dihydroxybenzoic acid	2	35
PA5	2,5-dihydroxybenzoic acid	2	30
PA6	2,6-dihydroxybenzoic acid	2	16
PA7	3,4-dihydroxybenzoic acid	2	60
PA8	3,5-dihydroxybenzoic acid	2	0
PA9	2-hydroxybenzoic acid	1	0
PA10	3-Hydroxybenzoic acid	1	0
PA11	4-Hydroxybenzoic acid	1	9
PA12	Benzoic acid	0	5
PA13	4-methoxybenzoic acid	0	0
PA14	3,4,5-trimethoxybenzoic acid	0	0
PA15	3,4,5-trifluorobenzoic acid	0	0

To further investigate the significance of the hydroxyl moieties on the activity of the GA and the other active phenolic acids, three additional compounds with either fluorides or methoxy groups rather than hydroxyl groups attached to their phenyl rings (4-methoxybenzoic acid, 3,4,5-trimethoxybenzoic acid and 3,4,5-trifluorobenzoic acid) (see Table 2) were tested for their ability to inhibit α-syn fibril formation. As expected, none of the three tested compounds in any of the three molar ratios tested (compound: α-syn 4:1, 2:1, and 1:1) could inhibit α-syn fibrillation (Figure 8). This finding provides additional support for the importance of the presence of the hydroxyl group in the phenyl ring.

## Discussion

The formation of amyloid aggregates has long been considered responsible for the pathogenesis of several neurodegenerative disorders (Taylor et al., 2002; Dorval and Fraser, 2007; Vekrellis et al., 2011). Although amyloidogenic proteins, such as α-syn, are usually found unfolded in their native state (Uversky, 2008; Fauvet et al., 2012), in which they are soluble and non-toxic, they can undergo misfolding resulting in the formation of insoluble aggregates (Seidler et al., 1996; Veldman et al., 1998; Irvine et al., 2008; Kahle, 2008; Stefanis, 2012). These aggregates deposit in various regions of the brain, constituting the main neuropathological feature of several neurodegenerative diseases such as PD, DLB, MSA and approximately the 50% of Alzheimer's disease cases. In the case of α-syn, pathological, biochemical, genetic and animal modeling studies provide compelling evidence that α-syn aggregation plays a pivotal role in the pathogenesis of PD and related synucleinopathies. As a consequence, the identification of compounds that can block or reverse the aggregation process of α-syn is considered a vital therapeutic strategy against these diseases.

To this end, we assessed the effect of the phenolic compound GA (3,4,5-trihydroxybenzoic acid) on α-syn aggregation and toxicity, and we established a structure-activity relationship for GA. By employing an array of biophysical and biochemical techniques, we showed that at a high concentration (100μM, represented by the molar ratio 4:1), GA exerts significant inhibitory effect on both α-syn fibrillation (Figures 1A,B) and oligomerization (Figures 2A,B), as well as seeded fibrillation of α-syn (Figure 4A). In accordance with these findings, TEM images revealed that α-syn in the presence of GA at high concentration only formed a few short, thin fibrils that had a fragmented appearance (Figure 1D) accounting for the low Th-S counts (Figure 1A) and the minor wavelength shift in the CR binding assay (Figure 1B). At lower GA concentrations (especially at 1:1 molar ratio), however, the ability of the GA to inhibit α-syn fibrillation was less dramatic and accompanied by a striking increase in the oligomeric content (Figure 2A), indicating GA may stabilize the oligomeric structure. Size exclusion chromatography combined with UV spectroscopy for detection of the incorporated GA in α-syn confirmed that GA binds to α-syn oligomers (Figure 6C). Furthermore, GA was able to disaggregate preformed α-syn fibrils (Figures 3A–C), generating species that possessed a decreased β-sheet content (Figure 3B) and were less toxic to the cells (Figure 3D). Previous studies have also shown that the flavonoid baicalein, as well as other antioxidant compounds inhibit α-syn fibrillation and disaggregate pre-aggregated fibrils (Zhu et al., 2004; Ono and Yamada, 2006). GA was also shown to alleviate α-syn aggregates associated-toxicity in neuroblastoma M17 cells (Figures 5A,B).

These results are in agreement with previous studies indicating that GA possesses anti-fibrillation, oligomer-stabilizing and neuroprotective properties against α-syn and Aβ cytotoxicity (Di Giovanni et al., 2010). Indeed, GA was shown to stabilize biotinylated Aβ_1–42_ oligomers (Levine et al., 2012) and to inhibit the *in vitro* conversion of low molecular weight Aβ_1–42_ protofibrils into fibrils by 50% (Di Giovanni et al., 2010). Additionally, GA was reported to block the fibrillation of α-syn *in vitro* and to suppress the ability of short α-syn fibrils to seed the aggregation of monomeric α-syn (Di Giovanni et al., 2010). Furthermore, GA exerts a neuroprotective effect on cells against the cytotoxicity of α-syn and Aβ_1–42_ (Di Giovanni et al., 2010), possibly due to the inhibition of the amyloid aggregation but potentially also due to the anti-inflammatory (Kim et al., 2011), anti-oxidant (Ban et al., 2008; Hong et al., 2012) and anti-apoptotic properties it possesses (Hong et al., 2012).

Based on our findings using both SEC and NMR, as well as previous reports, GA inhibits α-syn fibrillation not by interacting with the monomeric α-syn - similar to many other phenolic compounds (reviewed in Porat et al., 2006)-, and preventing it from polymerizing, but rather by stabilizing the structure of oligomeric α-syn (Figures 2B, 6C) which appears to be non-toxic (Figure 5). These findings are in accordance with previous studies indicating that polyphenolic compounds, such as baicalein, curcumin and epigallocatechin gallate (EGCG) induce the formation of soluble, non-toxic oligomers (Zhu et al., 2004; Masuda et al., 2006). From a therapeutic point of view, this represents a major advantage, as GA may not interfere with the physiological function of the monomeric α-syn (Wagner et al., 2013). It has been reported that phenolic compounds such as curcumin inhibit Aβ aggregation due to conformation-dependent binding (Yang et al., 2005). Consistent with this model of inhibition, GA also inhibited the seeding ability of short fibrillar α-syn. Although the presence of oligomers can be transient as they assemble into fibrils (Kaylor et al., 2005; Fink, 2006), there are studies revealing that in the presence of the polyphenolic compound EGCG, they can shift off-pathway from fibrillation (Ehrnhoefer et al., 2008). Indeed, EGCG was shown to promote the formation of less toxic oligomers that were exceedingly stable and incapable of contributing to the aggregation process (Ehrnhoefer et al., 2008). As it is also a phenolic compound, GA may interact with α-syn oligomers by a mechanism similar to that of EGCG, accelerating the formation of off-pathway oligomers and leading to the accumulation of non-toxic oligomers and the interruption of the fibrillation process.

To gain insight into the mechanism of action of GA and to establish a structure-function relationship for this phenolic acid, we evaluated the effect of various structurally similar phenolic acids on α-syn fibrillation. The results generated from this approach point toward a structure-related effect on α-syn fibrillation in which the better inhibitors were observed to have a greater number of -OH groups attached to the phenyl ring that were directly conjugated to the carboxylic acid arm (see Figure 8). In fact, the ranking of the anti-fibrillogenic potency of all the tested phenolic compounds could be represented as follows: trihydroxybenzoic acid > dihydroxybenzoic acid > monohydroxybenzoic acid = benzoic acid. This finding is in accordance with previous studies indicating that the potency of certain polyphenolic compounds to inhibit and disaggregate α-syn oligomers correlates with the number of vicinal -OH groups present on a single phenyl ring (Masuda et al., 2006; Caruana et al., 2011). The significance of the presence of the hydroxyl group on the phenyl ring was further stressed by the fact that the compounds that possess appendages other than hydroxyl groups on the phenyl rings failed to inhibit α-syn aggregation. At this point, it is more than an idle speculation to suggest that the inhibition capacity of the phenolic compounds tested is not only dependent on the number of hydroxyl moieties but also on their position and conjugation with respect to the benzoic acid appendage. Comparison of the two trihydroxybenzoic acids tested in this study, GA (3,4,5-trihydroxybenzoic acid) and 2,4,6-trihydroxybenzoic acid, indicates that although both compounds have a total of three -OH groups, the former is a much more potent inhibitor of α-syn fibrillation compared to the latter, strongly suggesting that it is not only the total number of -OH groups present in the molecule but also their position that is important. Additionally, comparison of the dihydroxybenzoic acids employed in the present study reveals that the presence of -OH groups in two consecutive positions and conjugated with the carboxyl group [e.g., 3,4-dihydroxybenzoic acid (60% inhibition) and 2,3-dihydroxybenzoic acid (44% inhibition)] renders the compounds more active compared to those that have hydroxyl groups at non-consecutive positions (e.g., 2,5-dihydroxybenzoic acid (30% inhibition) and 3,5-dihydroxybenzoic acid (0% inhibition) (see Figure 8 and Table 3). The importance of the 4-OH moiety is emphasized by the fact that of the three hydroxybenzoic acids tested, only 4-hydroxybenzoic acid could partially inhibit α-syn fibrillation. Among the dihydroxybenzoic acids, 3,4-dihydroxybenzoic acid was the most potent of all, inhibiting fibrillation by 60% (Table 3). Taken as a whole, these data show that the total number of -OH groups present in the molecule is important for the compound activity and that the position of the overall -OH groups also affects activity, with a hydroxyl group at position 4 appearing to play a significant role. Thus, the presence of three vicinal hydroxyl groups, as in 3,4,5-trihydroxy benzoic acid (GA), or the three homo-vicinal groups, as in 2,4,6-trihydroxy benzoic acid, with one -OH group at position 4, renders the compounds able to inhibit α-syn fibrillation to a great extent.

Given that all the compounds tested in this study have a phenyl ring in their structures, the phenyl structure itself is clearly not sufficient to inhibit α-syn aggregation. Consequently, the inhibition ability of such compounds must be achieved by the presence of additional -OH groups attached to the phenyl ring. The conjugated hydroxyl groups with the carboxylic acid appendage can easily form quinone structures (see Figure S4). It is worth mentioning that quinone formation has been previously reported as the potential mechanism of inhibition of α-syn fibrillation by the phenolic compound baicalein (Zhu et al., 2004). The formation of such oxidized derivatives can increase the stability of the compound binding to the α-syn fibrils (Taniguchi et al., 2005), possibly accounting for the increased inhibitory potency of the compounds with more -OH groups present in their structures. Quinones are likely to interact with the aromatic residues of α-syn, interfering with their π−π stacking (Porat et al., 2006; Ebrahimi and Schluesener, 2012; Hamley, 2012). Moreover, quinones have a planar orientation that allows them to align with the hydrophobic groove of α-syn fibrils, which possess an in-register organization of side chains in the regular cross-β-sheet structure (Porat et al., 2006; Ebrahimi and Schluesener, 2012; Hamley, 2012). Therefore, the inhibition of α-syn fibrillation by GA and 2,4,6-trihydroxybenzoic acid could be attributed to the combined properties of their three -OH groups and quinone-forming structures. Based on these results, we propose a plausible model to interpret the inhibitory and disassembling effects of GA. Two dominant ligand-protein interactions contribute to inhibitor potency, (a) the dipole-dipole interactions of the quinone and (b) the dipole-dipole interactions of the diquinone group. The most potent inhibitors, with 3,4,5-trihydroxy and 2,4,6-trihydroxy substitution patterns (GA and 2,4,6-trihydroxy benzoic acid, respectively), (see Figure S4c), enable both hydrophobic and dipole-dipole interactions. The π−π stacking is known to play a role in the binding affinity of inhibitors to aggregates, increasing the potential for quinone formation of each compound generally leading to increased binding affinity and, therefore, increased inhibitor potency. Consistent with this interpretation, ortho- and meta-hydroxybenzoic acid, as well as benzoic acid (see Figure S4f) showed no inhibition effect compared to their para- and ortho-paradihydroxy (Figure S4a) counterparts due to the inability of the former to form quinone-like ligands (Figures S4f, d).

Although monocyclic compounds such as phenolic acids were previously shown to be very poor inhibitors of aggregation (Reinke and Gestwicki, 2007), later studies revealed that monocyclic compounds could suppress amyloid assembly by interacting with oligomers, stabilizing their structure and conformation, thus inhibiting the transition to β-sheet structures (Levy-Sakin et al., 2009). It is therefore likely that phenolic acids, especially GA, act in a similar way, interacting with oligomers and stabilizing their structure through the -OH groups, preventing the transition to β-sheet-rich structures. This may explain the increased oligomeric content detected at low GA concentrations and the low β-sheet content of α-syn species generated in the presence of GA.

In summary, we conducted a systematic study of the effect of GA on α-syn fibrillation, oligomerization and toxicity, and we concluded that GA inhibits α-syn fibrillation and toxicity by stabilizing the non-toxic oligomeric structure of α-syn through oligomer binding. Our study points toward a structure-related inhibition, with the number of -OH moieties and their position around the phenyl ring playing a fundamental role. Based on the above experimental results, we propose a model to interpret the inhibitory and disassembling effects of GA derivatives on α-syn aggregation (see Figure S5). α-Syn aggregation is a sequential process in which unstructured α-syn monomers undergo conformational transition and re-ordering to form oligomers and then finally amyloid fibrils (routes a → b → c → d, Figure S5). The analysis of the results obtained from this study shows that GA derivatives can prolong the nucleation process, suggesting that these scaffolds can bind to unstructured α-syn oligomers and prevent protein association (Figure S5, route a → f) and/or slow down the conformational transition to structured oligomers, the precursors of amyloid fibrils. GA derivatives interact with α-syn monomers and/or oligomers through π −π stacking, leading to the formation of an energy barrier that prevents the association of α-syn molecules. Meanwhile, GA derivatives through their respective quinone oxidation products could induce structural disruption to the local β-sheet of α-syn fibrils via strong binding to the β-sheet groove regions of α-syn fibrils, leading to fibril disaggregation (d → e). It should be noted that, due to the hydrophobic aromatic nature and planar structure of quinones, they might interact with α-syn fibrils by relatively nonspecific dipole-dipole interactions with β-sheet-rich side chains. This binding mode implies that quinones could have a general inhibitory effect on the aggregation of α-syn.

### Conflict of interest statement

The authors declare that the research was conducted in the absence of any commercial or financial relationships that could be construed as a potential conflict of interest.
