# Supplementary material for: Structure activity relationship of phenolic acid inhibitors of α-synuclein fibril formation and toxicity
Source: Front Aging Neurosci. 2014 Aug 5;6:197. doi: 10.3389/fnagi.2014.00197 (PMC4122169; doi:10.3389/fnagi.2014.00197)
Supplement: Supplementary file 1 [file Presentation1.PDF]

Supplementary Material

Structure Activity Relationship of Phenolic Acid inhibitors of α-Synuclein Fibril Formation and Toxicity

Mustafa Taleb Ardah[1,], Katerina E. Paleologou[1], Guohua Lv[2], Salema Begum Abul Khair[1], Abdullah KazimAl Kendi[1], Saeed Tarek Minhas[1], Taleb H. Al-Tel[3], Abdulmonem A. Al-Hayani[4], Mohammed Emdadul Haque[1], David Eliezer[2] and Omar M. A. El-Agnaf [1,5]*

[1]Department of Biochemistry, College of Medicine and Health Science, United Arab Emirates University, Al Ain, United Arab Emirates.
[2]Department of Biochemistry, Weill Cornell Medical College, New York, NY, USA
[3]College of Pharmacy and Sharjah Institute for Medical Research, University of Sharjah, Sharjah, UAE.
[4]Department of Anatomy, Faculty of Medicine, King Abdulaziz University, Jeddah, Saudi Arabia.
[5]Faculty of Medicine, King Abdel Aziz University, Jeddah, Saudi Arabia.

*** Correspondence:** Omar M. A. El-Agnaf, Department of Biochemistry, College of Medicine and Health Science, United Arab Emirates University, Al Ain, United Arab Emirates.
o.elagnaf@uaeu.ac.ae

1. Supplementary Figures and Tables

1.1. Suplementary Figures

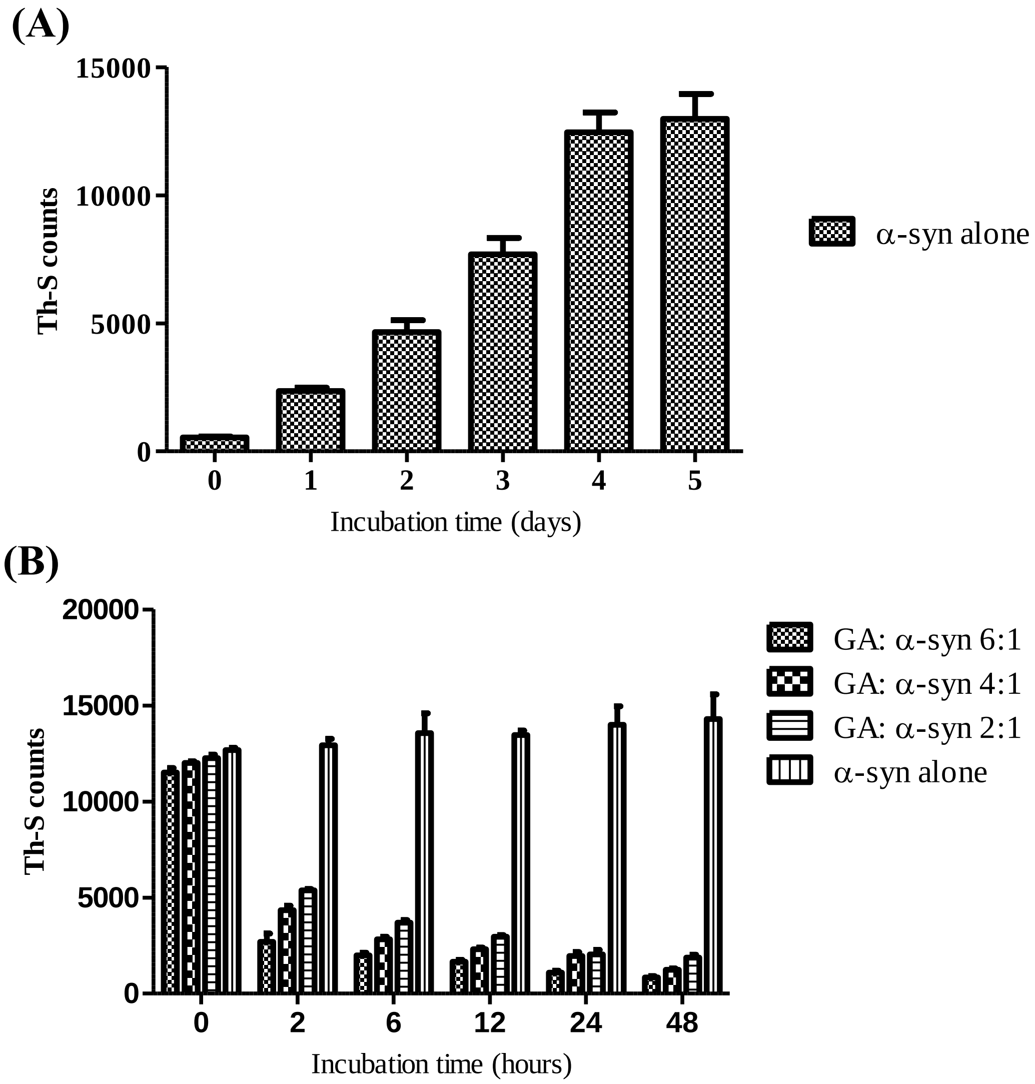

Supplementary Figure 1. GA disaggregates preformed α-syn fibrils (after reaching plateau). A. Samples of α-syn (25 µM) were incubated alone for 5 days with continuous shaking at 37°C. Fibril formation was estimated by Th-S fluorescence. The assay was performed in triplicate (average of triplicate measurement ± standard deviation). B. Samples of aggregated α-syn were incubated for 48 hours at 37°C in the absence or presence of various concentrations of GA (GA: α-syn 6:1, 4:1, 2:1). The fibril content was then measured by the Th-S binding assay. The assays were performed in triplicate (average of triplicate measurements ± standard deviations).

(A)

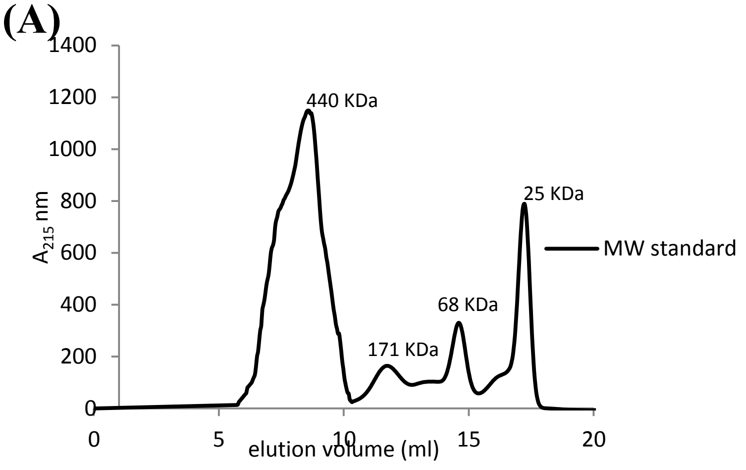

(B)

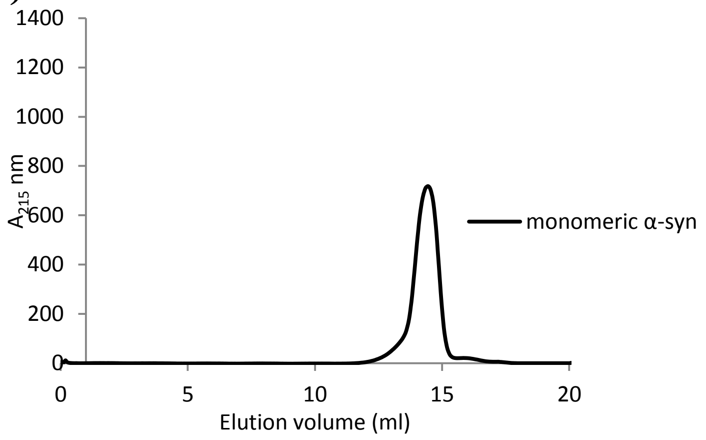

Supplementary Figure 2. Size Exclusion Chromatography: A: Gel-filtration profile of MW standard containing Ferritin 440 KDa, Aldolase 171 KDa, Abmumin 68 KDa and Chymotrypsinogen A 25 KDa using Superdex 200 column at 0.1 ml/min flow rate (0.5 ml/fraction). B: Gel-filtration profile for monomeric α-syn using Superdex 200 column at 0.1 ml/min flow rate (0.5 ml/fraction).

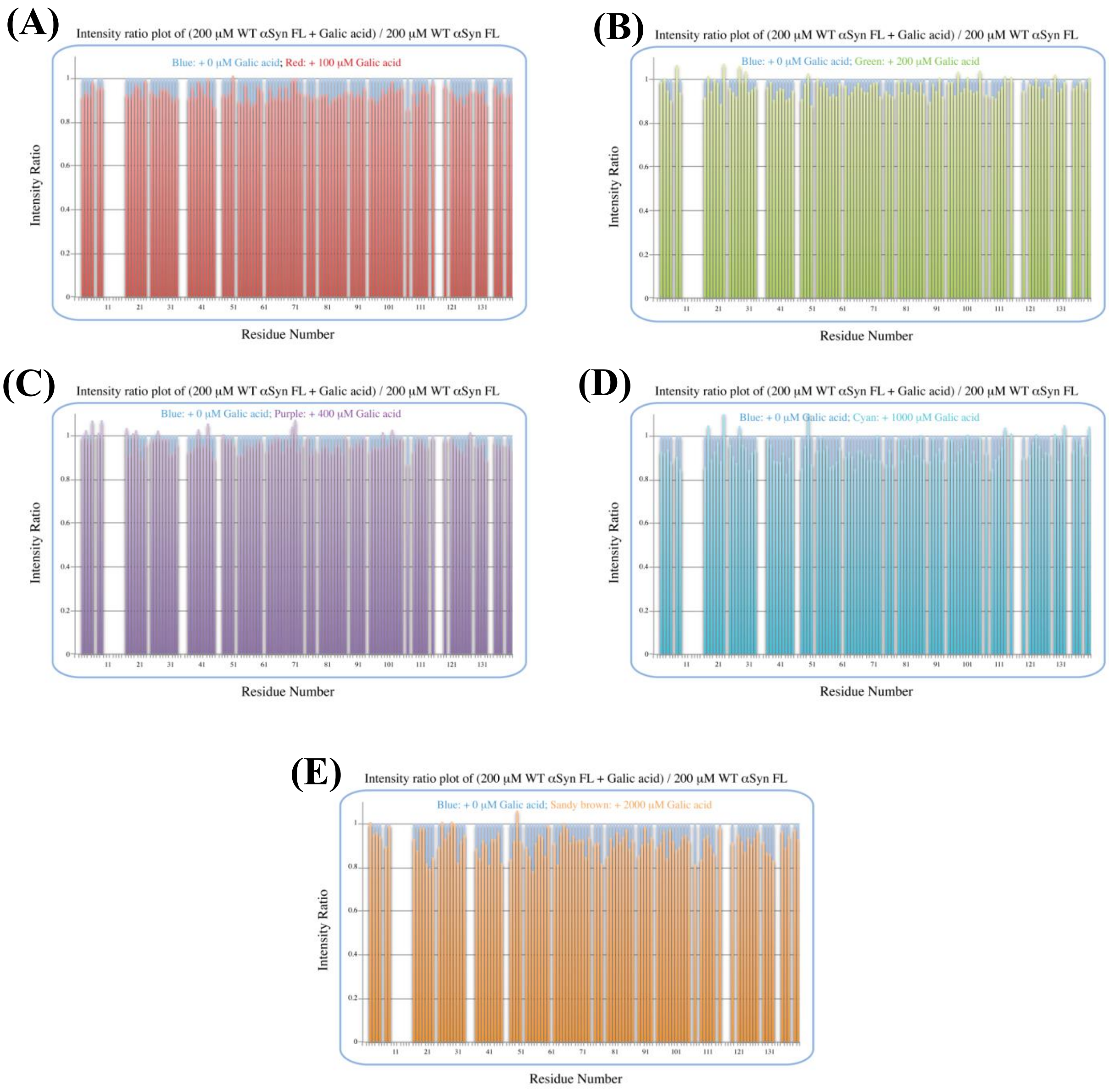

Supplementary Figure 3. Analysis of GA binding to monomeric α-syn by NMR spectroscopy**.** Ratios of NMR resonance intensities from proton-nitrogen (HSQC) correlation spectra in the presence of increasing ratios of GA: α-syn (A - 0.5:1, B - 1:1, C - 2:1, D - 5:1, E - 10:1) to intensities observed in the absence of GA. The absence of any significant intensity changes in the presence of GA again confirms the lack of an interaction between GA and monomeric α-syn.

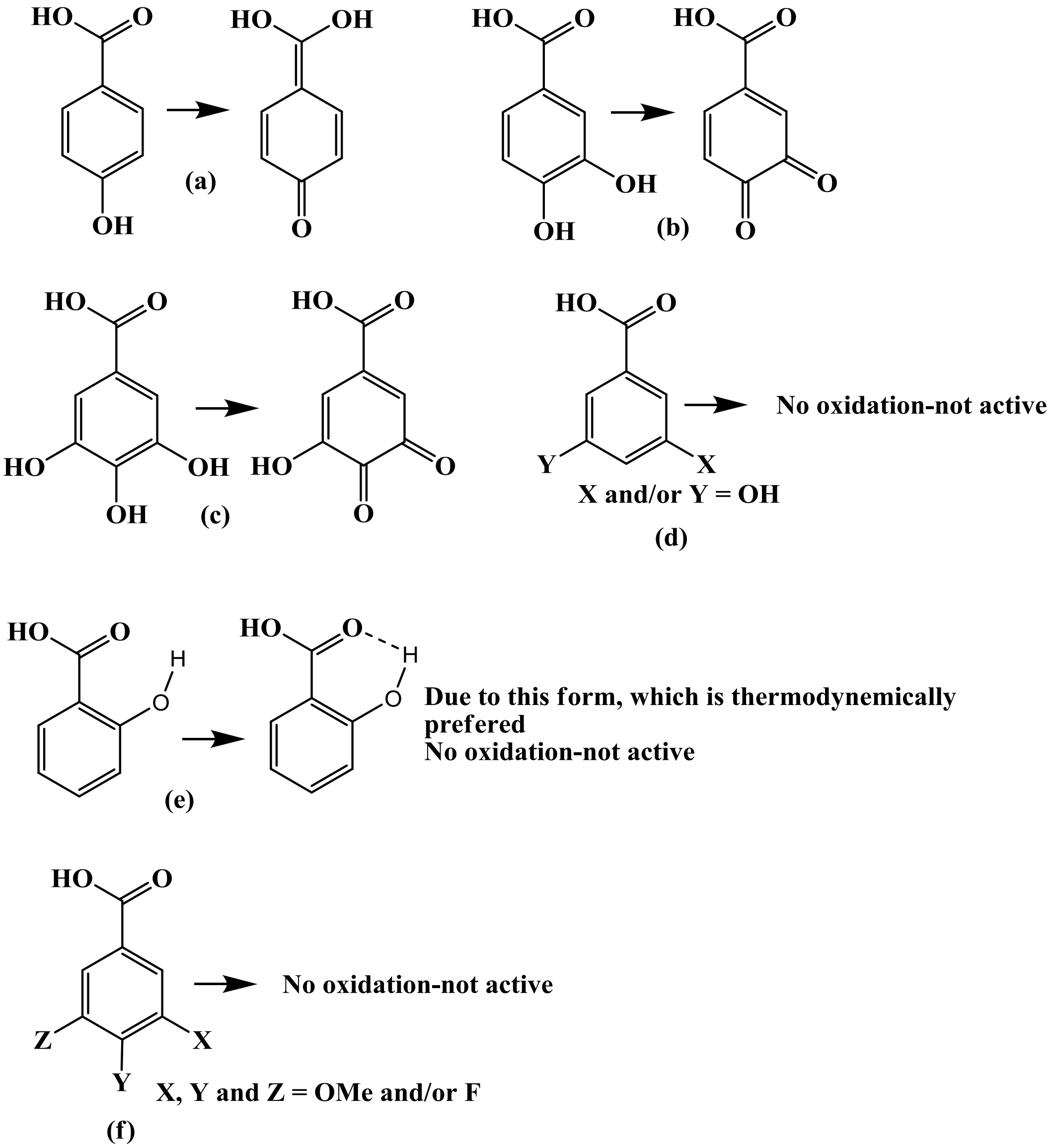

Supplementary Figure 4. **Mechanistic insight for the mechanism of quinone formation.**

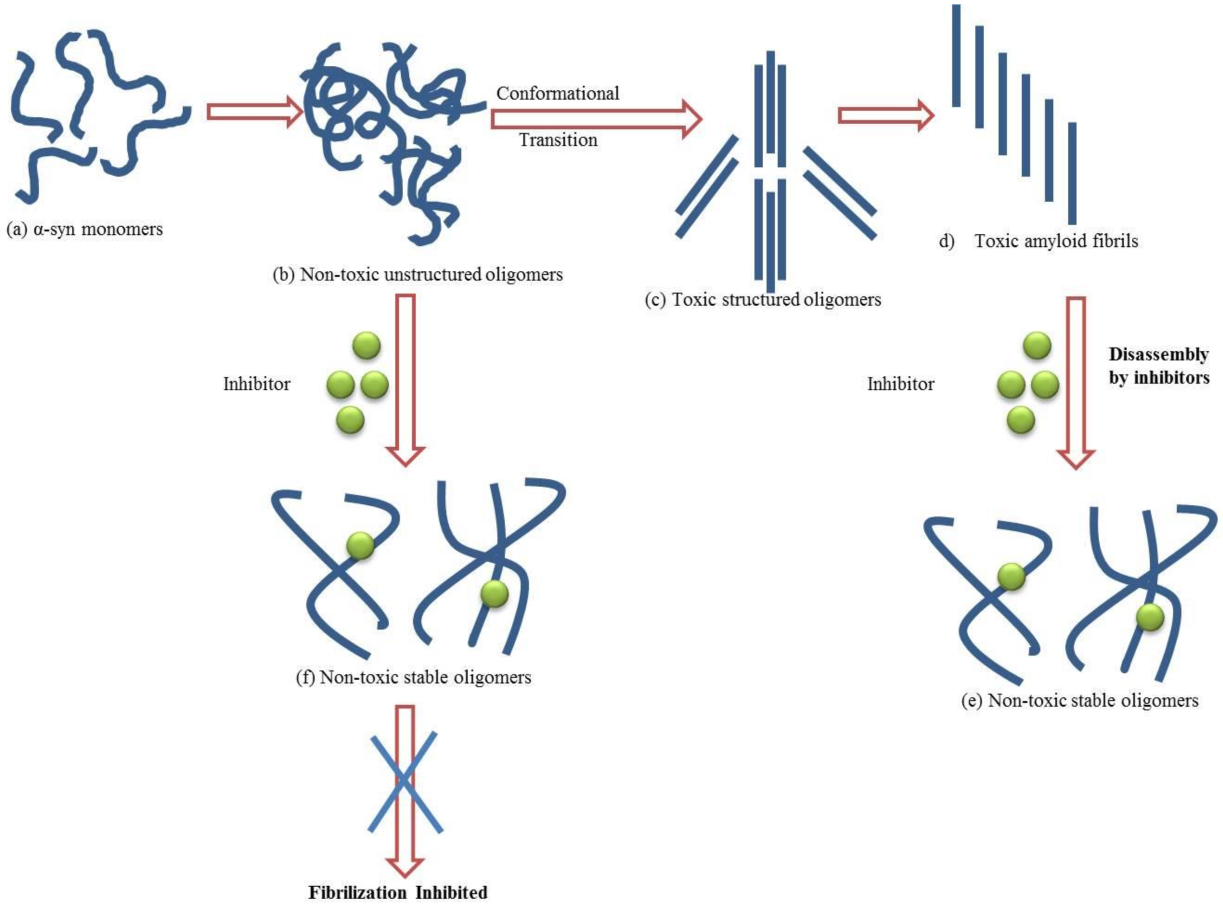

Supplementary Figure 5. Schematic model for the antiaggregation and disassembly effect of GA derivatives on α-syn.
